# Hyperthermic Laser Ablation of Recurrent Glioblastoma Leads to Temporary Disruption of the Peritumoral Blood Brain Barrier

**DOI:** 10.1371/journal.pone.0148613

**Published:** 2016-02-24

**Authors:** Eric C. Leuthardt, Chong Duan, Michael J. Kim, Jian L. Campian, Albert H. Kim, Michelle M. Miller-Thomas, Joshua S. Shimony, David D. Tran

**Affiliations:** 1 Brain Laser Center, Department of Neurological Surgery, Washington University in St. Louis School of Medicine, St. Louis, Missouri, 63110, United States of America; 2 Department of Neurological Surgery, Washington University in St. Louis School of Medicine, St. Louis, Missouri, 63110, United States of America; 3 Center for Innovation in Neuroscience and Technology, Washington University in St. Louis School of Medicine, St. Louis, Missouri, 63110, United States of America; 4 Department of Biomedical Engineering, Washington University in St. Louis School of Medicine, St. Louis, Missouri, 63110, United States of America; 5 Department of Mechanical Engineering and Material Sciences, Washington University in St. Louis School of Medicine, St. Louis, Missouri, 63110, United States of America; 6 Department of Chemistry, Washington University in St. Louis School of Medicine, St. Louis, Missouri, 63110, United States of America; 7 Neuro-Oncology Program, Division of Oncology, Department of Medicine, Washington University in St. Louis School of Medicine, St. Louis, Missouri, 63110, United States of America; 8 Division of Neuroradiology, Mallinckrodt Institute of Radiology, Washington University in St. Louis School of Medicine, St. Louis, Missouri, 63110, United States of America; 9 Division of Neuro-Oncology, Lillian S. Wells Department of Neurological Surgery, McKnight Brain Institute, The University of Florida College of Medicine, Gainesville, Florida, 32610, United States of America; German Cancer Research Center (DKFZ), GERMANY

## Abstract

**Background:**

Poor central nervous system penetration of cytotoxic drugs due to the blood brain barrier (BBB) is a major limiting factor in the treatment of brain tumors. Most recurrent glioblastomas (GBM) occur within the peritumoral region. In this study, we describe a hyperthemic method to induce temporary disruption of the peritumoral BBB that can potentially be used to enhance drug delivery.

**Methods:**

Twenty patients with probable recurrent GBM were enrolled in this study. Fourteen patients were evaluable. MRI-guided laser interstitial thermal therapy was applied to achieve both tumor cytoreduction and disruption of the peritumoral BBB. To determine the degree and timing of peritumoral BBB disruption, dynamic contrast-enhancement brain MRI was used to calculate the vascular transfer constant (*K*^*trans*^*)* in the peritumoral region as direct measures of BBB permeability before and after laser ablation. Serum levels of brain-specific enolase, also known as neuron-specific enolase, were also measured and used as an independent quantification of BBB disruption.

**Results:**

In all 14 evaluable patients, *K*^*trans*^ levels peaked immediately post laser ablation, followed by a gradual decline over the following 4 weeks. Serum BSE concentrations increased shortly after laser ablation and peaked in 1–3 weeks before decreasing to baseline by 6 weeks.

**Conclusions:**

The data from our pilot research support that disruption of the peritumoral BBB was induced by hyperthemia with the peak of high permeability occurring within 1–2 weeks after laser ablation and resolving by 4–6 weeks. This provides a therapeutic window of opportunity during which delivery of BBB-impermeant therapeutic agents may be enhanced.

**Trial Registration:**

ClinicalTrials.gov NCT01851733

## Introduction

Glioblastoma (GBM) is the most common and lethal malignant brain tumor in adults [1]. Despite advanced treatment, median survival is less than 15 months, and fewer than 5% of patients survive past 5 years [2, 3]. Effective treatment options for recurrent GBM remain very limited and much of research and development efforts in recent years have focused on this area of greatly unmet needs. Up to 90% of recurrent tumors develop within the 2–3 cm margin of the primary site and are thought to arise from microscopic glioma cells that infiltrate the peritumoral brain region prior to resection of the primary tumor [4, 5]. Therefore elimination of infiltrative GBM cells in this region likely will improve long-term disease control.

Inadequate CNS delivery of therapeutic drugs due to the blood brain barrier (BBB) has been a major limiting factor in the treatment of brain tumors. The presence of contrast enhancement on standard brain MRI qualitatively reflects a disrupted state of the BBB. For this reason, drug access to the viable contrast enhanced tumor rim is likely significantly higher than to the peritumoral region, which usually does not have contrast enhancement [6, 7]. Evidence supporting this hypothesis came from studies in which drug levels of cytotoxic agents were sampled in tumors and the surrounding brain tissue at the time of surgery or autopsy. Drug concentrations were at the highest in the enhancing portion of tumors, and then rapidly decreased up to 40 fold lower by 2–3 cm distance from the viable tumor edge [8–10]. Overall, these observations suggest that the BBB and its integrity negatively correlate with delivery and potentially therapeutic effects of BBB impermeant drugs.

To circumvent the BBB problem in local drug delivery, recent approaches have focused on bypassing it. A previously described method is the use of Gliadel, a polymer wafer impregnated with the chemotherapeutic agent carmustine (BCNU) and placed intra-operatively in the resection cavity to bypass the BBB. This approach resulted in a statistically significant but modest survival advantage in both newly diagnosed and recurrent GBM [11–13]. The modest benefit of Gliadel could be due to the short duration of drug delivery as nearly 80% of BCNU is released from the wafer over a period of only 5 days [14]. This observation further supports the notion that the BBB is critical to chemotherapy effect. However, Gliadel is not widely utilized as it requires an open craniotomy and can impair wound healing. Another approach of bypassing the BBB is the convection-enhanced delivery system in which a catheter is surgically inserted into the tumor to deliver chemotherapy [15]. This procedure requires prolonged hospitalization to maintain the external catheter to prevent serious complications and as a result has not been used extensively.

The role of hyperthermia in inducing BBB disruption has been previously described in animal models of CNS hyperthermia. In a rodent model of glioma, the global heating of the mouse’s head to 42°C for 30 minutes in a warm water bath significantly increased the brain concentration of a thermosensitive liposome encapsulated with adriamycin chemotherapy [16]. To effect more locoregional hyperthermia, retrograde infusion of a saline solution at 43°C into the left external carotid artery in the Wistar rat reversibly increased BBB permeability to Evans-blue albumin in the left cerebral hemisphere [17]. In another approach, neodymium-doped yttrium aluminum garnet (Nd:YAG) laser-induced thermotherapy to the left forebrain of Fischer rats resulted in loco-regional BBB disruption as evidenced by passage of Evans blue dye, serum proteins (e.g. fibrinogen & IgM), and the chemotherapeutic drug paclitaxel for up to several days after thermotherapy [18]. The effect of hyperthermia on the BBB of human brain has not been examined.

Here we describe an approach to induce sustained, local disruption of the peritumoral BBB using MRI-guided laser interstitial thermal therapy, or LITT. The biologic effects and correlation with MRI findings of LITT have been studied in both animal and human models since the development of LITT over twenty years ago. A well-described zonal distribution of histopathological changes with corresponding characteristic MR imaging findings centered on the light-guide track replace the lesion targeted for thermal therapy. The central treatment zone shows development of coagulative necrosis with complete loss of normal neurons or supporting structures immediately following therapy, corresponding to hyperintense T1-weighted signal intensity relative to normal brain [19–22]. The peripheral zone of the post-treatment lesion is characterized by avid enhancement with intravenous gadolinium contrast agents, which peaks several days following thermal therapy and persists for many weeks after the procedure. Gadolinium contrast enhancement in the brain following LITT is due to leakage of gadolinium contrast into the extravascular space across a disrupted BBB [20–23]. The perilesional zone of hyperintense signal intensity of FLAIR-weighted images develops within 1–3 days of thermal treatment and persists for 15–45 days [22].

We demonstrate that in addition to cytoreductive ablation of the main recurrent tumor, hyperthermic exposure of the peritumoral region resulted in localized, lasting disruption of the BBB as quantified by dynamic contrast-enhanced MRI (DCE-MRI) and serum levels of brain-specific enolase (BSE), thus providing a therapeutic window of opportunity for enhanced delivery of therapeutic agents.

## Materials and Methods

### Patient selection

Adult patients (age ≥ 18 years) with unequivocal evidence of recurrent bevacizumab-naïve, histologically confirmed GBM were screened for eligibility to participate in a protocol approved by the Internal Review Board of Washington University in St. Louis School of Medicine (S2 File). Written informed consent was obtained from each participant prior to any study-related activity. Patients included in this report were the first 20 patients enrolled in a larger ongoing pilot phase II trial (ClinicalTrials.gov identifier number NCT01851733), in which a total of 40 patients with recurrent GBM will be enrolled. Patients with prior diagnosis of a WHO grade II or III gliomas were eligible if the recurrent tumor had radiographic characteristics of a GBM, WHO grade IV. For these subjects to be included a biopsy was obtained immediately prior to LITT and subsequent pathologic analysis must have confirmed secondary GBM, WHO grade IV. General characteristics that make the lesion(s) favorable to treatment include the following: (1) the lesion(s) is (are) supratentorial and accessible from a cephalad approach (i.e., top one third of the head), (2) the lesion(s) is (are) unilateral, (3) the lesion(s) is (are) relatively well circumscribed, (4) the volume of lesion(s) can be encompassed by two 3-cm cylinders (i.e., 2 treatment trajectories), (5) a safe trajectory can be established relative to functional structures (i.e., eloquent cortex and corticospinal tract), and (6) the patient’s body habitus can fit into the bore of the MRI.

### Study design

**Fig 1:**
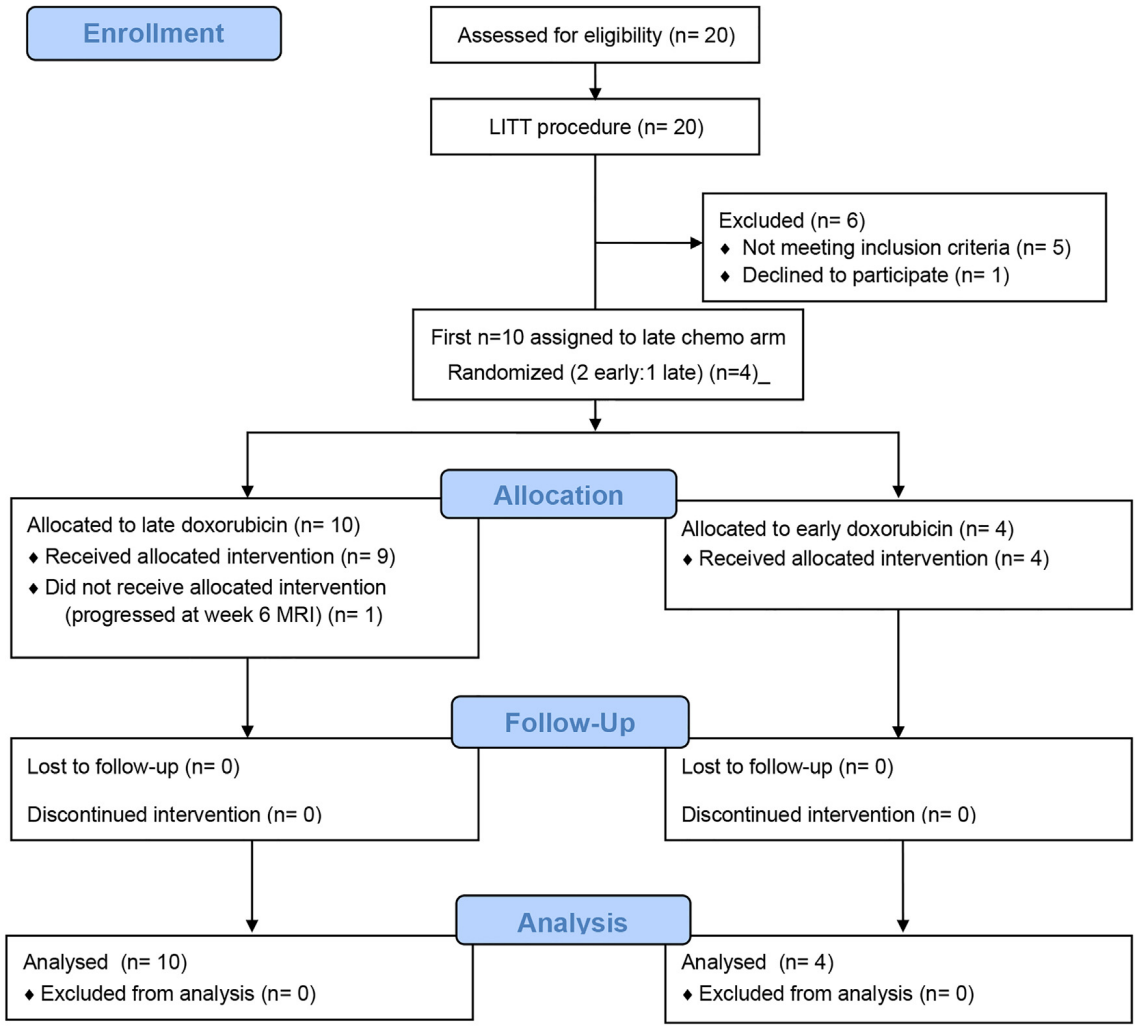
CONSORT flow diagram of the BBB disruption measurement portion of the pilot phase 2 study involving the first 20 enrolled patients. Early or Early doxorubicin: Treatment started within 1 week after LITT. Late or Late doxorubicin: Treatment started at 6 weeks after LITT.

The pilot study has 2 main objectives: 1) To determine the safety of LITT in patients with recurrent GBM and spatiotemporal MR imaging correlates and serum biomarkers of peritumoral BBB disruption after LITT; and 2) To determine whether treatment with the BBB-impermeant chemotherapy agent doxorubicin dosed at 20mg/m^2^ IV weekly for 6 doses during the window of post-LITT BBB disruption is safe and will improve local disease control compared to when the same agent given during the window of intact BBB. The first 10 patients were assigned to the late (starting 6 weeks after LITT) doxorubicin treatment arm so that MRI and serum biomarker measurements can be performed without potential confounding effects of chemotherapy. The next 30 patients are randomized at the ratio of 2 to 1 to either early (starting within 1 week after LITT) or late doxorubicin treatment to achieve the final distribution of 20 patients in each arm (S2 File). Non-evaluable patients due to any reason will be replaced. Data collected in the first 10 weeks after LITT from the first 20 enrolled patients were focused on the biophysical parameters of measurements of BBB disruption, which is independent from the second objective and provides the foundation for this report (Fig 1) (S1 File). Patients underwent a pre-LITT baseline DCE-MRI and biomarker measurement within 48 hours prior to LITT. Patients underwent post-LITT baseline DCE-MRI and biomarker measurement within 48 hours after LITT. Subsequent DCE-MRIs were performed at weeks 2, 4, 6, and 10 after LITT. Biomarker measurements were performed weekly for 6 weeks and at week 10 after LITT.

### MRI-guided Laser Thermal Ablation Therapy (LITT)

The Neuroblate system, Monteris, Inc., was used to deliver LITT of GBM. LITT is a minimally invasive laser surgery currently cleared by the FDA for interstitial thermal treatment of brain lesions with 1064 nm lasers [24–27]. LITT employs a small incision in the scalp and skull, through which a thin laser probe is inserted and guided by MR imaging to the core of a tumor mass where it delivers hyperthermic ablation with the maximal temperature in tumor core reaching 60–70°C resulting in coagulative necrosis, while the temperature decreases to 40–45°C in the peritumoral region [27]. Trajectories are chosen to maximize lesion ablation and minimize the number of passes. Live intra-procedural repetitive measurements of a T1-weighted 2-dimensional-FLASH sequence provide temporally sensitive thermometry measurements necessary to create controlled and conformal lesions.

### DCE-MRI

Dynamic contrast-enhancement (DCE) [28] is a method that relies on dynamically measuring the changes in T1-weighted images following the administration of an intravascular contrast agent. Using pharmacokinetic modeling this method can estimate the vascular transfer constant (*K*^*trans*^*)* [29]. *K*^*trans*^ describes the ability of contrast to move from the intravascular compartment to the extracellular extravascular compartment and thus provide a quantitative measure of the degree of BBB leakage [29].

### MRI Protocol

Standard of care imaging with added DCE-MRI was obtained within 48 hours prior to, within 48 hours after and then at weeks 2, 4, 6, and 10 after LITT. All patients were scanned on the same Siemen’s Avanto 1.5T MRI (Erlanger, Germany) identically using a tumor follow up standard of care imaging protocol that including anatomical imaging sequences (T1-weighted pre and post-contrast, T2-weighted images, FLAIR images) augmented with rapid T1-weighted DCE protocol (3D gradient echo, TR/TE = 4.8/2.4ms, matrix 256x256x44, voxel size 1.5x1.5x4mm, temporal resolution of 1 volume per 10 seconds for a total of 6 minutes or 36 volumes after the administration of Multihance (Gadobenate Dimeglumine, Bracco) 0.1 mmol/kg injected at a rate of 5 mL/s.

### Data Analysis

The area of the FLAIR signal abnormality was measured in the single slice in which it was maximal in extent. The area measurement was performed using the region of interest (ROI) tool in Analyze (version 10, Mayo Clinic, Rochester, MN). For the DCE-MRI analysis ROIs were defined on the enhancing ring surrounding the ablated tumor on the DCE-MRI images using post-contrast T1-weighted image and FLAIR images for guidance. The ROI was selected on the portion of the ring that demonstrated maximal contrast enhancement and were within 1cm of the margin of ablation. In some subjects there was no visible contrast enhancement on the immediate post-operative scan and in those cases no measurement was made at that time point. The outlined ROIs were transferred to all the other DCE time point measurements within each dataset. Signal intensity *vs*. time curves was generated as the average within the ROIs. To avoid partial volume effect, an arterial input function (AIF) was obtained from one voxel in the center of the middle cerebral artery for each dataset. The MR signal intensities were then converted to contrast agent concentrations as described by Kallehauge et al., [30] using reported pre-contrast T_1_ and contrast agent relaxivity.

Using the data from the first 5 subjects we performed a data-driven Bayesian model selection process looking at 4 different models: the standard Tofts model [28], extended Tofts model [28], compartment tissue uptake model [31], and two-compartment exchange model [32]. With the quality of our data the standard Tofts model consistently gave the highest posterior probability among the different models. This manifested as more robust and stable parameter estimation as compared to the other DCE models. Therefore, quantitative pharmacokinetic parameters (*K*^*trans*^ and *υ*_e_) were estimated for each dataset by applying a standard Tofts Model [28]:

$$C_{t}\left(t\right)=K^{trans}\times \int_{0}^{t}C_{a}\left(\tau \right)\times e^{-\left(K^{trans}/v_{e}\right)\times \left(t-\tau \right)}d\tau$$

where *C*_t_(*t*) is the tissue contrast agent concentration *vs*. time curve obtained at the enhanced region following the administration of contrast agent bolus, *C*_t_(*t*) is the plasma concentration of the contrast agent in the capillary inlet of the tissue and is approximated by the measured upstream AIF following the standard DCE-MRI analysis approach [33], K^trans^ is the forward volume transfer constant (from vascular to extravascular extracellular space), which is a direct reflection of capillary permeability, and *υ*_e_ is the extracellular extravascular volume fraction.

All pharmacokinetic modeling was performed using a custom written Bayes Data-Analysis Toolkit (http://bayesiananalysis.wustl.edu). The posterior probabilities for all the model parameters were computed by an application of Bayes’ Theorem with Markov-chain Monte Carlo simulation. Initial values for all the parameters were sampled from the prior probability for each parameter. Uniformly distributed prior probabilities bounded by appropriate physiological ranges were assigned to all of the parameters. The prior ranges selected are 0–6.0 min^-1^ for *K*^*trans*^, and 0–1 for *υ*_e_. In the current study we focused on the values of the *K*^*trans*^. Computations were carried out on Dell PowerEdge R900 server (Dell, Inc. Round Rock, TX), Further details about Bayesian parameter estimation are given in Lee et al [34].

### Measurement of serum biomarker levels of BBB disruption

Serum levels of BSE were measured using an ELISA kit (Alpco) per the manufacturer’s instructions.

### Statistical analysis

For serum BSE optimization analysis, 2-sided Student T test was used. Pearson correlation coefficients were calculated between the time courses of *K*^*trans*^, serum BSE levels, and the area of FLAIR abnormality for each of the subjects. Since the MRI measurements were only performed at a few time points the curves were interpolated into a smooth curve using piecewise cubic Hermite interpolation.

## Results

### Patient characteristics and treatment course

Twenty bevacizumab-naïve patients with suspected recurrent GBM were enrolled in the first phase of the larger pilot phase II study. Fifteen patients (#1, 4, 5, 7, 9, 10, 12–20) were diagnosed histologically with primary GBM, WHO grade IV. All received standard concurrent radiation and temozolomide chemotherapy per the Stupp protocol [2, 3], except for Patient #9, who received radiation alone without concurrent temozolomide, followed by standard adjuvant temozolomide. Patient #4 withdrew consent. Patients #2, 6, and 11 and Patients 3 and 8 were initially diagnosed with a WHO grade III and grade II glioma, respectively, and subsequently developed recurrent tumor with radiographic appearances of probable transformed or secondary GBM. However needle biopsy immediately prior to LITT failed to demonstrate secondary GBM, which rendered these subjects ineligible and DCE-MRI and serum BSE data were not obtained. Several patients had participated in prior clinical trials for newly diagnosed GBM. All recurrent tumors were approximately 3 cm or less at the longest dimension (Table 1).

**Table 1:** Patient Baseline Demographics and Characteristics. TMZ/RT: Stupp protocol of 60 Gy radiotherapy plus concurrent 75mg/m2 daily temozolomide. Doxorubicin treatment: Timing of 20mg/m^2^ IV weekly doxobubicin treatment after LITT. Early = Starting within 1 week after LITT; Late = Starting at 6 weeks after LITT.

PtN°	Age Range	Initial Diagnosis	Molecular Biomarkers	Tumor Location	TMZ/RT	Eligible?	Doxorubicin Treatment
1	50–60	GBM	Unmethylated MGMT	Left temporal	Yes	Yes	Late
2	40–50	Astrocytoma WHO grade III	1p, 19q intact; IDH1 wild-type	Left parietal	Yes	No (GBM unconfirmed)	N/A
3	60–70	Astrocytoma WHO grade II	1p, 19q intact; IDH1 wild-type	Left parietal	Yes	No (GBM unconfirmed)	N/A
4	50–60	GBM	Methylated MGMT	Right parietal	Yes	No (withdrew consent)	N/A
5	60–70	GBM	Unmethylated MGMT; EGFRvIII	Left temporal	Yes	Yes	Late
6	40–50	Astrocytoma WHO grade III	1p, 19q intact; IDH1 wild-type	Left temporal	Yes	No (GBM unconfirmed)	N/A
7	40–50	GBM	Methylated MGMT; EGFRvIII	Right frontal	Yes	Yes	Late
8	30–40	OligoastrocytomaWHO grade II	1p, 19q intact; IDH1 R132H	Left insular	Yes	No (GBM unconfirmed)	N/A
9	50–60	GBM	Methylated MGMT; EGFRvIII	Left thalamic	No (RT only)	Yes	Late
10	60–70	GBM	Unmethylated MGMT	Left parietal	Yes	Yes	Late
11	40–50	Astrocytoma WHO grade III	1p, 19q intact; IDH1 R132H	Right frontal	Yes	No (biopsy, LITT not performed)	N/A
12	60–70	GBM	Unmethylated MGMT	Left frontal	Yes	Yes	Late
13	60–70	GBM	MGMT methylation unknown	Right frontal	Yes	Yes	Late
14	60–70	GBM	Methylated MGMT; EGFRvIII	Right temporal	Yes	Yes	Late
15	60–70	GBM	Unmethylated MGMT	Right frontal	Yes	Yes	N/A (progressed at week 6)
16	50–60	GBM	Unmethylated MGMT; EGFRvIII	Left frontal	Yes	Yes	Early
17	50–60	GBM	Unmethylated MGMT	Right parieto-occipital	Yes	Yes	Early
18	50–60	GBM	Unmethylated MGMT; EGFRvIII	Left parietal	Yes	Yes	Late
19	70–80	GBM	Methylated MGMT; EGFRvIII	Left frontal	Yes	Yes	Early
20	60–70	GBM	Methylated MGMT; EGFRvIII	Right parietal	Yes	Yes	Early

Pre-procedure, DCE-MRI was performed on the eligible subjects. The average *K*^*trans*^ value in the tumors before treatment was 0.22 1/min (N = 12), (standard deviation 0.15 1/min, range 0.05–0.66 1/min).

LITT procedure was well tolerated in all of the first 20 patients in this study. The average length of post-LITT hospitalization stay was 1.73 days. The median stay was 1 day with a range of 1–5 days. No severe adverse events deemed related to the study treatments were observed. Two patients required a short course of oral steroid treatment after LITT without significant complications. Of the 14 eligible patients, Patients #12 developed local progression at the time of the week-10 MRI scan; Patient #15 developed multifocal progression at the time of the week-6 MRI scan, was removed from study treatment and did not receive late doxorubicin treatment. The rest of other eligible patients exhibited no radiographic or clinical evidence of disease progression during the 10-week time frame of the study.

### Quantitative measurement of LITT-induced peritumoral BBB disruption by DCE-MRI

**Fig 2:**
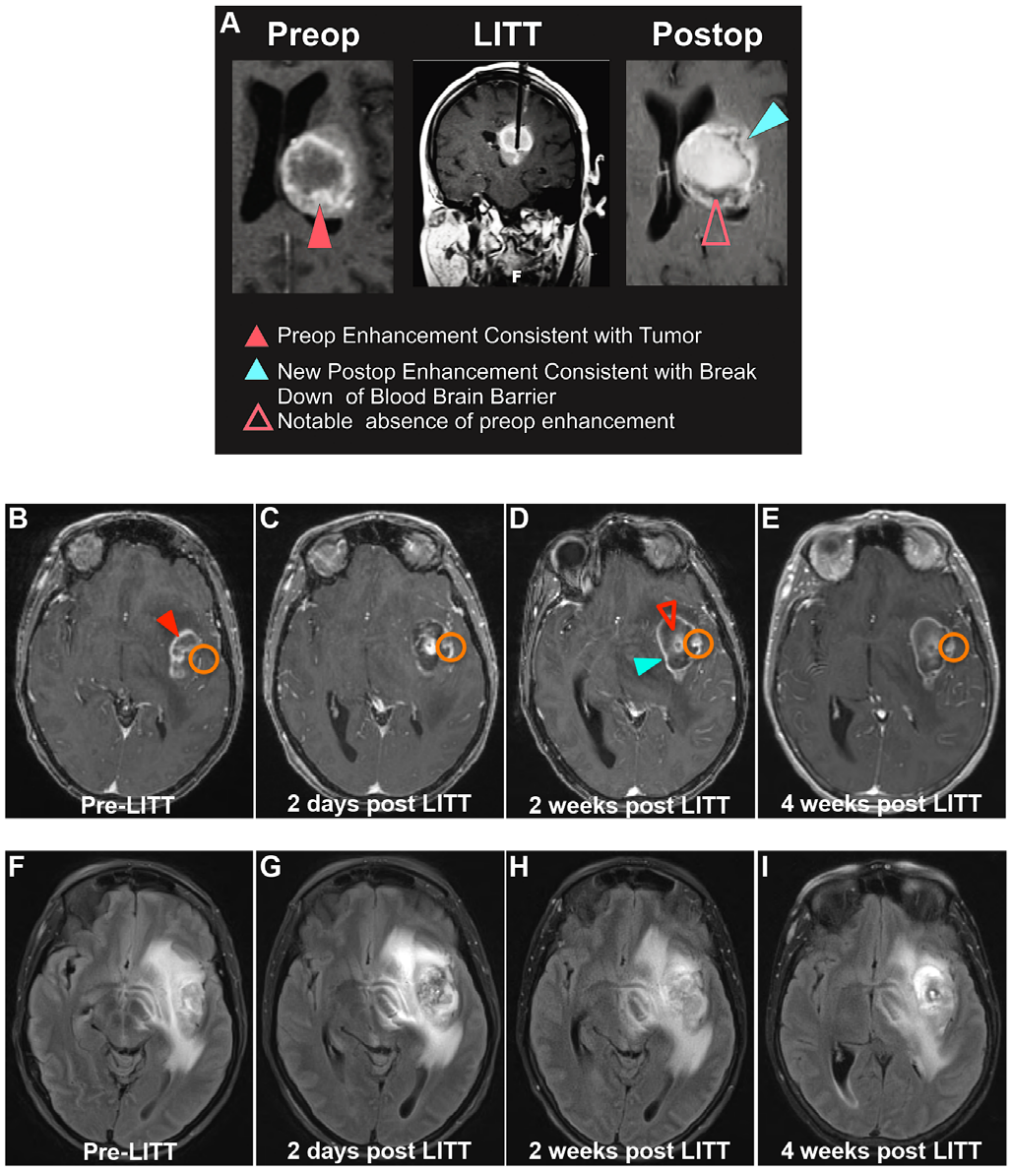
Radiographic appearances of post-LITT changes. (**A**) A woman with a left thalamic GBM treated with LITT underwent axial and coronal T1-weighted post-contrast enhanced MR images of the brain pre-LITT, during LITT and 48 hours post LITT. (**B-I**) A woman with a left insula GBM underwent axial T1-weighted post-contrast enhanced (**B-E**) and axial FLAIR-weighted (**F-I**) MR images of the brain pre-LITT and within 48 hours post LITT, 2 weeks post LITT, and 4 weeks post LITT. In both cases, the enhancing tumor (solid red arrowheads in **A** and **B**) is replaced by a central zone of T1-weighted signal hyperintensity (open red arrowheads in **A** and **D**) and a faint, new discontinuous rim of enhancement extending beyond the original tumor associated enhancing rim (solid blue arrowheads in **A** and **D**). The rim of contrast enhancement intensifies at 2 weeks post LITT (**D**) and remains stable at 4 weeks post LITT (**E**). Perilesional edema evaluated on FLAIR-weighted images is slightly increased between the pre-treatment (**F**) and immediate post-treatment (**G**) images, increases to a maximum point on the 2-week post-treatment images (**H**) and improves slightly by the 4-week images (**I**). The orange circles denote a representative ROI used to calculate temporal progression of *K*^*trans*^ after LITT

Brain MRI obtained within 48 hours following LITT showed the targeted tumor replaced by a post-treatment lesion corresponding to the volume of treated tissue on intraoperative thermometry maps. The post-treatment lesion lost the original rim of tumor-associated contrast enhancement and instead demonstrated central hyperintense T1-weighted signal compared to the pre-treated tumor and normal brain and a faint, newly developed discontinuous rim of peripheral contrast enhancement extending beyond the original tumor-associated enhancing rim (Fig 2A). These findings are consistent with a loss of viable tumor tissue caused by LITT, thus achieving an effective cytoreduction similar to open surgical resection. Of note, the rim of new peripheral contrast enhancement persisted for at least the next 28 days (Fig 2B–2E). Perilesional edema qualitatively evaluated on FLAIR-weighted images increased from pretreatment imaging at week 2 and persisted at week 4 following LITT (Fig 2F–2I). Perilesional edema decreased on subsequent MRI examinations. These findings qualitatively indicate that peritumoral BBB is disrupted by LITT and that the disruption peaks within approximately 2 weeks after the procedure.

**Fig 3:**
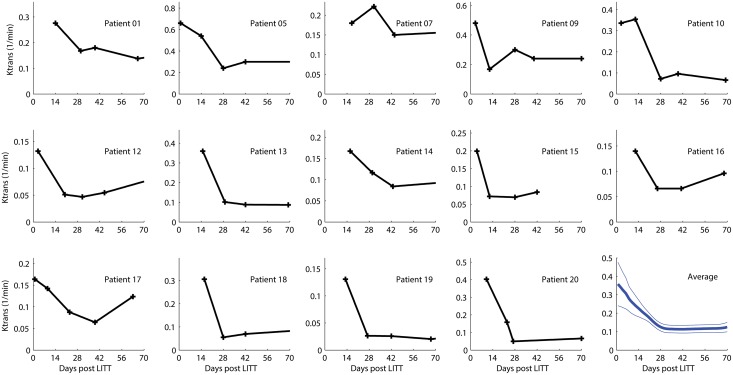
Peritumoral BBB disruption induced by LITT as measured by DCE-MRI. *K*^*trans*^ for each of the 14 eligible subjects in the study as a function of time in days from the LITT procedure. In all subjects the *K*^*trans*^ is highly elevated in the first few days after the procedure and then progressively decreases out to approximately the 4-week time point. This is best illustrated in the bottom right blue graph, which is an average of the 14 subject curves.

Fig 3 demonstrates the *K*^*trans*^ time curves for our cohort of patients. In all subjects the *K*^*trans*^ in the ROIs within the enhancing ring around the ablated tumor is highly elevated in the first few days after the procedure and then progressively decreases at approximately the 4-week time point. The bottom right subplot in Fig 3 is an average of the *K*^*trans*^ time courses from all the subjects with adjacent curves indicating the plus and minus one standard error of the mean curves. This figure demonstrates the peak *K*^*trans*^ value immediately after the LITT procedure with persistent elevation out to about 4 weeks. Radiographically, persistent contrast enhancement and FLAIR hyperintensity were observed well past 6 weeks and in many cases more than 10 weeks later. Several patients had recurrent tumor by radiographic criteria (increasing size of the edema and enhancing area around the tumor site) and these patients also demonstrated a corresponding increase in the *K*^*trans*^ value. These recurrences occurred after the 10-week mark and thus were not included in Fig 3. Importantly no difference in the pattern of *K*^*trans*^ tracing was consistently observed between the 10 patients receiving late doxorubicin treatment and the 4 patients receiving early doxorubicin treatment. In summary, these results indicate that the peritumoral BBB disruption as measured by *K*^*trans*^ peaked immediately after LITT and persisted above baseline for an additional 4 weeks.

### Quantitative measurement of LITT-induced peritumoral BBB disruption by serum BSE biomarker

**Fig 4:**
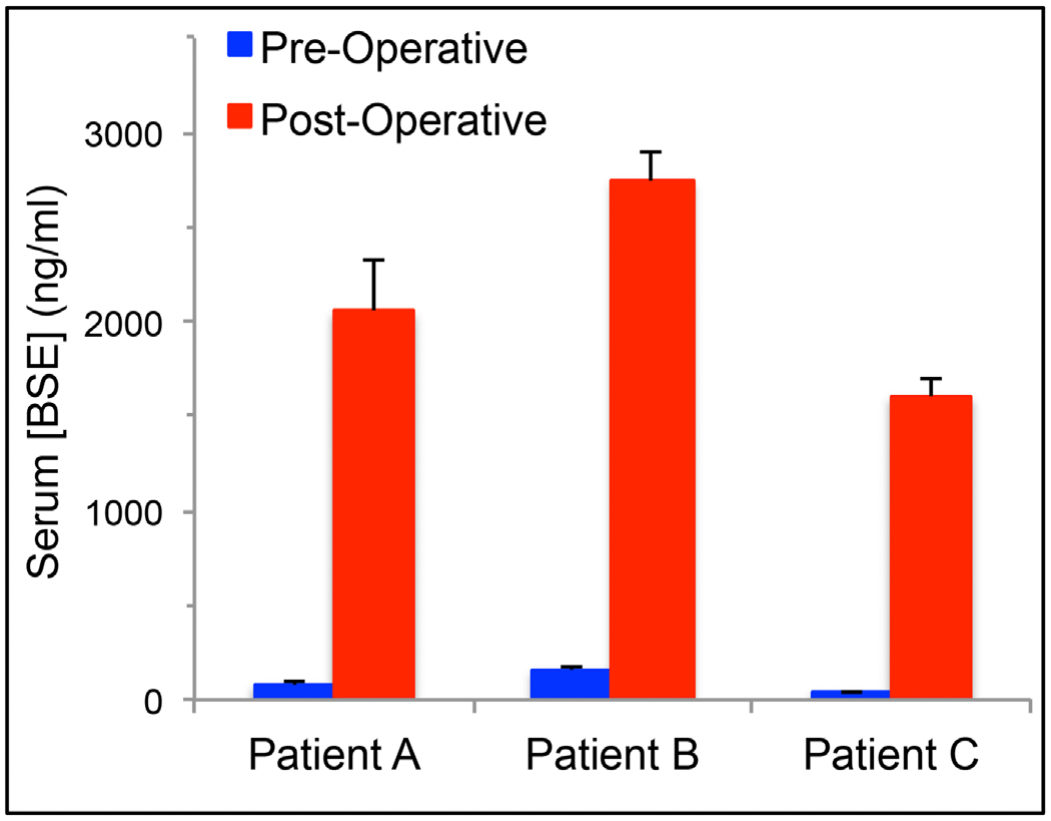
Optimization of the BSE ELISA assay for measuring BBB disruption. Serum concentrations of BSE before and after open craniotomy for surgical debulking in 3 subjects (A, B, and C) with a low-grade glioma, WHO grade II. *p<0.05.

We next sought another, independent method to quantify the degree of BBB disruption by measuring serum levels of the brain specific factor BSE that might be released into the circulation due to the increased BBB permeability using highly sensitive assays such as ELISA. This method has been validated for quantitative measurement of BBB disruption induced by several forms of brain injuries including surgery, traumatic brain injury, cerebrovascular accident and multiple sclerosis [35–39]. To optimize the ELISA assay for BSE, we collected sera from 3 patients with a newly diagnosed low-grade (WHO grade 2) glioma before and after their planned craniotomy and surgical resection, and determined serum concentrations of BSE. WHO grade 2 gliomas were chosen for the optimization because as they are generally non-contrast enhanced tumors on brain MRI, tumor-associated BBB is relatively intact and consequently, serum concentrations of brain-specific factors are predicted to be low pre-operatively and to then rise post-operatively due to the BBB compromise from the surgery. Serum BSE concentrations were low prior to surgery and then, as predicted, consistently increased after open craniotomy and tumor resection, thus indicating that this method had adequate sensitivity in detecting changes in serum levels of BSE due to disruption of the BBB (Fig 4).

**Fig 5:**
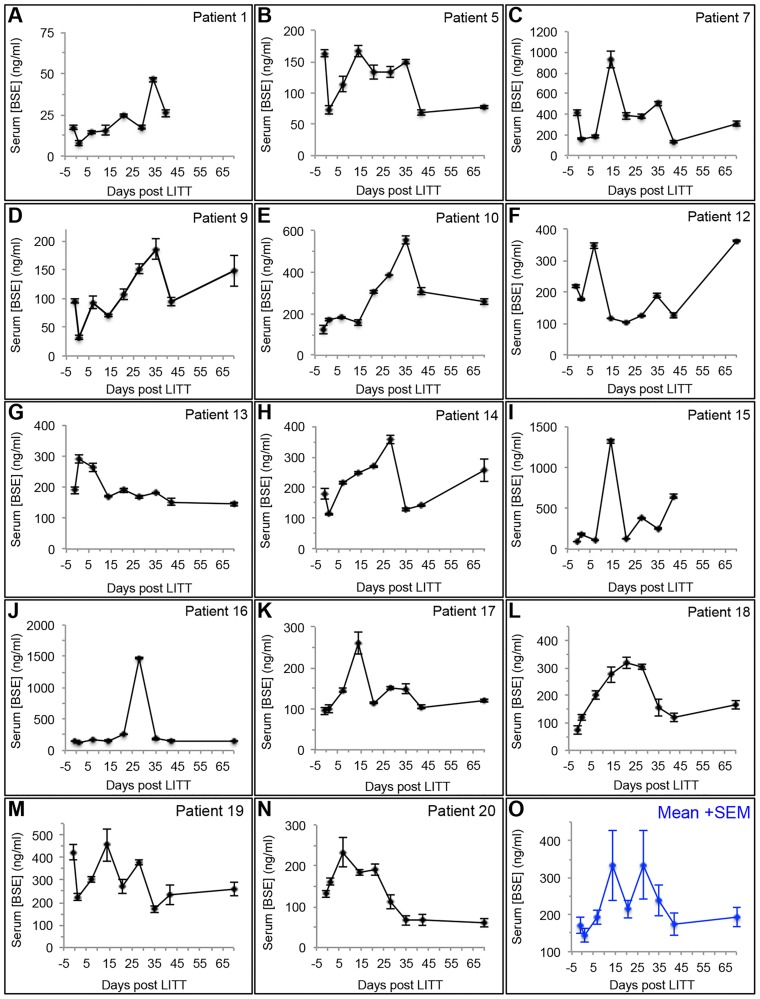
BBB disruption induced by LITT as measured by serum biomarkers. Serum concentrations of BSE for each of the 14 evaluable subjects in the study (**A-N**) and as the mean + SEM (**O**) as a function of time in days from the LITT procedure. In 7/14 subjects, serum BSE levels slightly decreased immediately after LITT, then in 13/14 subjects, serum BSE levels rose shortly after LITT, peaked between 1–3 weeks after LITT, and then decreased by the 6-week time point. In Patient #12, serum BSE concentration increased at week 10 coincident with an increased *K*^*trans*^ at the same time point, consistent with a recurrent tumor as demonstrated on diagnostic MR imaging. Patient #15’s serum BSE concentration began to rise by week 4, consistent with early multifocal recurrent disease as demonstrated on diagnostic MR imaging.

We next determined concentrations of BSE in sera obtained from the 14 evaluable LITT patients within 48 hours prior to LITT, within 48 hours after LITT and weekly thereafter for 6 weeks, and then at week 10 after LITT, and compared them to *K*^*trans*^ values in the same patient. Serum BSE concentrations demonstrated a steady rise shortly after LITT, and although there were fluctuations among patients, in most serum BSE concentrations peaked by 2 to 3 weeks and then gradually declined over the subsequent 2–3 weeks (Fig 5). Compared to *K*^*trans*^, peak concentrations of BSE had more temporal variations and were delayed by up to 1 to 2 weeks. Serum sample for Patient 1 was not obtained at week 10 since an amendment to the protocol to allow serum collection at this time point was not yet approved by the local IRB. Similar to the *K*^*trans*^ results, the serum BSE concentration in Patient #12 rose rapidly after week 6, which coincided with the emergence of contrast-enhanced recurrent disease demonstrated on the brain MRI at week 10. Patient #15 had an early rise in serum BSE concentrations and a small increase in *K*^*trans*^ at week 4 (Fig 5), and was also found to have contrast-enhanced multifocal recurrences at week 6 and therefore the patient was removed from study treatment and serum sample was not obtained at week 10. Again, we did not observe consistent differences in the serum BSE profile between the late and early doxorubicin treatment arms. In summary, when combined with the DCE-MRI measurements, these results provide further confirmation that besides allowing for effective tumor cytoreduction LITT induces disruption of the peritumoral BBB that persists up to 4 to 6 weeks after the procedure.

### Correlation Analysis

The Pearson correlation values between the *K*^*trans*^ measurements and the serum BSE values was *r =* 0.28 with standard deviation (stdev) of 0.52. The correlation calculation resulted from averaging of two different groups of subjects. Subjects that had an early rise in their BSE serum values had a higher positive correlation with the DCE-MRI *K*^*trans*^ results. Subjects with a delayed rise in their BSE serum values had a lower and, in some cases, negative correlation with the *K*^*trans*^ values. The Pearson correlation of the area of FLAIR signal abnormality was *r =* 0.25 (stdev = 0.61) with the *K*^*trans*^, and *r =* 0.18 (stdev = 0.59) with the BSE serum values.

## Discussion

LITT is a minimally invasive neurosurgical technique that achieves effective tumor cytoreduction of brain tumors using a laser to deliver hyperthermic ablation. Here we have demonstrated that an unexpected, potentially useful effect of LITT is its ability to also disrupt the BBB in the peritumoral region that extends outwards 1–2 cm from the viable tumor rim. Importantly, the disruption persists in all 14 evaluable, treated patients for up to 4 weeks after LITT as measured quantitatively by DCE-MRI and up to 6 weeks as measured by serum levels of the brain-specific factor BSE. These observations indicate that after LITT there is a window during which enhanced local delivery of therapeutic agents into the desired location (i.e. peritumoral region) can potentially be achieved.

In all of the patients in this series, the peaks of serum concentrations of BSE showed wider variations and were delayed from several days to 1–2 weeks following the peak of BBB disruption as measured by *K*^*trans*^. The wider variations and delay of BSE concentrations lead to relatively low correlation coefficients between the two parameters and could be explained by: 1) the higher data point resolution for the serum values versus DCE-MRI values (weekly versus biweekly, respectively); 2) interval physiologic breakdown of thermally ablated tissue coupled with subsequent diffusion and equilibration between the intracranial and peripheral compartments; and 3) high inter-tumor heterogeneity among patients resulting in a wide variation in the rates at which ablated tissues of different compositions are broken down and released into the circulation. Whether these differences may be in part due to tumor-related factors such as IDH1/2 mutations and MGMT promoter methylation is unclear due to the small number of subjects. More importantly, both methods showed that the peritumoral BBB disruption induced by LITT was temporary, decreasing soon after peaking and being resolved by 4–6 weeks in most patients. In addition, although no significant difference in all the BBB measurement parameters was observed between the early and late doxorubicin treatment arms, the number of evaluable subjects was too small to allow generalization at this time. Nevertheless, we did not notice any credible cause to differ data processing between these 2 subject groups until additional data become available later in the larger study suggesting otherwise. Overall, our present data demonstrating a dual application of LITT to achieve cytoreduction and to induce reversible disruption of the peritumoral BBB should allow for the reexamination of drugs that have not demonstrated a survival advantage in prior studies or are predicted to be ineffective in primary or metastatic brain tumors because of their poor BBB penetration (e.g. monoclonal antibodies or highly hydrophilic compounds) despite possessing considerable anti-cancer activity in vitro and/or in extracranial tumors.

Whether the 4–6 week duration of BBB disruption after LITT is long enough to be therapeutically meaningful will need to be determined prospectively in future studies. In the case of Gliadel, direct delivery of BCNU into the resection cavity over a short period of 5 days was sufficient to result in a modest survival benefit for both recurrent and newly diagnosed GBM [14] when compared to BCNU administered systemically. Therefore the significantly longer duration of BBB disruption induced by LITT would be predicted to be adequate for enhanced drug delivery and clinical benefits when the right therapeutic agents are utilized.

In addition to the role LITT could play for enhanced local delivery of therapeutic agents, there is also the possibility that this approach could have important immunological consequences. The persistent elevation of BSE after LITT in the peripheral circulation indicates that proteins are being continually released outside the immune-privileged compartment of the CNS. With the laser ablation of the tumor, it is reasonable to assume the tumor specific proteins are also being released into systemic circulation. Whether this enhanced presentation of tumor antigens and neoantigens to the immune system could facilitate the body’s tumor-specific immune response remains to be determined, but is quite an intriguing line of inquiry for future investigation.

## Supporting Information

S1 File CONSORT 2010 Checklist. (PDF)

S2 File Study Protocol. (PDF)
