# Supplementary material for: Hyperthermic Laser Ablation of Recurrent Glioblastoma Leads to Temporary Disruption of the Peritumoral Blood Brain Barrier
Source: PLoS One. 2016 Feb 24;11(2):e0148613. doi: 10.1371/journal.pone.0148613 (PMC4766093; doi:10.1371/journal.pone.0148613)
Supplement: S2 File — (PDF) [file pone.0148613.s002.pdf]

A Pilot Study of Using MRI-Guided Laser Heat Ablation to Induce Disruption of the Peritumoral Blood Brain Barrier to Enhance Delivery and Efficacy of Doxorubicin in the Treatment of Recurrent Glioblastoma Multiforme

Washington University School of Medicine
Division of Oncology
660 South Euclid Avenue, Campus Box 8056
St. Louis, MO 63110

Protocol#: 201305148
Version Date: 05/28/2014

**Principal Investigator:**	David D. Tran, M.D., Ph.D.<br>Phone: (314) 362-8967<br>E-mail: dtran@dom.wustl.edu

**Sub-Investigators**	**Modality**
Eric C. Leuthardt, M.D.	Neurosurgery
Joshua S. Shimony, M.D., Ph.D.	Neuro-Radiology
Gavin Dunn, MD, PhD	Neurosurgery
Michael Chicoine, MD	Neurosurgery
Albert Kim, MD, PhD	Neurosurgery
Ralph Dacey, MD	Neurosurgery
Joshua Dowling, MD	Neurosurgery
Gerald Linette, MD, PhD	Medical Oncology
Gregory Zipfel, MD	Neurosurgery
Keith Rich, M.D.	Neurosurgery
Clifford Robinson, MD	Radiation Oncology
Joseph Simpson, MD, PhD	Radiation Oncology
Jiayi Huang, MD	Radiation Oncology
Esther Liu, PhD	Biostatistician

**Study Drug:**	Doxorubicin
**IND #:**	118584 EXEMPT
**Clinical Trials.gov #:**	NCT01851733

CONFIDENTIAL
The information contained in this document is regarded as confidential and, except to the extent necessary to obtain informed consent, may not be disclosed to another party unless law or regulations require such disclosure. Persons to whom the information is disclosed must be informed that the information is confidential and may not be further disclosed by them.

A Pilot Study of Using MRI-Guided Laser Heat Ablation to Induce Disruption of the Peritumoral Blood Brain Barrier to Enhance Delivery and Efficacy of Doxorubicin in the Treatment of Recurrent Glioblastoma Multiforme

Protocol Revision History

**Initial Approval Version**	**06/25/2013**
**Amendment #1 Version**	**10/01/2013**
**Amendment #2 Version**	**01/08/2014**
**Amendment #3 Version**	**05/28/2014**

A Pilot Study of Using MRI-Guided Laser Heat Ablation to Induce Disruption of the Peritumoral Blood Brain Barrier to Enhance Delivery and Efficacy of Doxorubicin in the Treatment of Recurrent Glioblastoma Multiforme

SCHEMA

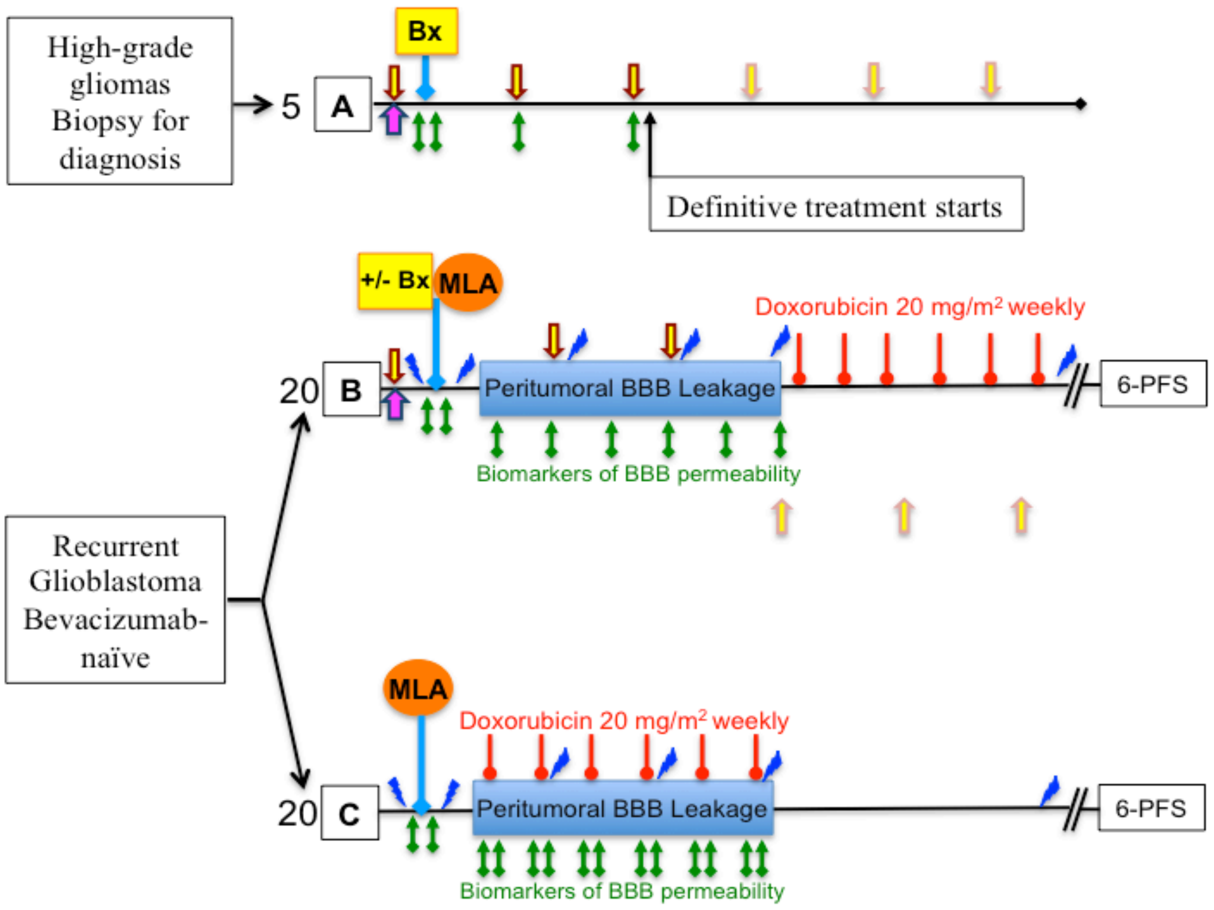

- = **DSC-MRI**
- MLA = **MRI-guided laser heat ablation**
- Bx = **Biopsy only, no MLA or heating**
- = **Dendritic cell (DC) harvest**
- = **Peripheral blood monocyte (PBMC) collection**
- = **Optional PBMC collection if no significant cytopenias**
- = **Biomarker laboratory (prior to and after MLA then weekly x 6 weeks)**
- 6-PFS = **6 month progression-free survival rate**

Glossary of Abbreviations

AE	Adverse event
ALT (SGPT)	Alanine transaminase (serum glutamate pyruvic transaminase)
ANC	Absolute neutrophil count
ALP	Alkaline phosphatase
ASCO	American society for clinical oncology
AST (SGOT)	Aspartate transaminase (serum glutamic oxaloacetic transaminase)
BBB	Blood brain barrier
B-HCG	Beta human chorionic gonadotropin
BSE	Brain-specific enolase
CBC	Complete blood count
CBV	Cerebral blood volume
CFR	Code of Federal Regulations
CNS	Central nervous system
CR	Complete remission
CRF	Case report form
CST	Central standard time
CT	Computed tomography
CTCAE	Common Terminology Criteria for Adverse Events
CTEP	Cancer Therapy Evaluation Program
DNA	Deoxyribonucleic acid
DCE-MRI	Dynamic contrast-enhanced magnetic resonance imaging
DSC-MRI	Dynamic susceptibility contrast enhanced magnetic resonance imaging
DSM	Data and Safety Monitoring
EDTA	Ethylenediaminetetraacetic acid
ELISA	Enzyme-linked immunosorbent assay
EMEA	European Agency for Evaluation of Medicinal Products
ESA	Erythrocyte stimulating agent
FDA	Food and Drug Administration
FLAIR	Fluid-attenuated inversion recovery
FWA	Federal wide assurance
GBM	Glioblastoma multiforme
GCP	Good Clinical Practice
G-CSF	Granulocyte colony stimulating factor, filgrastim (Neupogen)
GFAP	Glial fibrillary acidic protein
GGT	Gamma-glutamyl transpeptidase
GM-CSF	Granulocyte/macrophage colony stimulating factor, sargramostim, (Leukine, Prokine)
HHS	Department of Health and Human Services'
HIV	Human Immunodeficiency Virus
HRPO	Human Research Protection Office (IRB)
ICH	International Conference on Harmonization
IND	Investigational New Drug

IRB	Institutional Review Board
IV	Intravenous (i.v.)
LD	Longest diameter
LDH	Lactate dehydrogenase
MLA	MRI-guided Laser Ablation (Monteris Neuroblate)
MMSE	Mini-mental state examination
MRI	Magnetic resonance imaging
NCCN	National Cancer Center Network
NCI	National Cancer Institute
NIH	National Institutes of Health
OHRP	Office of Human Research Protections
PD	Progressive disease
PI	Principal investigator
PR	Partial response
QASMC	Quality Assurance and Safety Monitoring Committee
qPCR	Quantitative polymerase chain reaction
RBC	Red blood cell (count)
ROI	Region of interest
RR	Response rate
SAE	Serious adverse event
SCC	Siteman Cancer Center
SD	Stable disease
SOC	Standard of care
TMZ	Temozolomide
TSH	Thyroid stimulating hormone
TTP	Time to progression
ULN	Upper limit of normal
UPN	Unique patient number
VEGF	Vascular endothelial growth factor
WBC	White blood cell (count)
WHO	World Health Organization
WLN	Within limit of normal

Table of Contents

SCHEMA ... 3
1.0 BACKGROUND AND RATIONALE ... 8
1.1 Glioblastoma Multiforme (GBM) ... 8
1.2 Penetrating the Blood Brain Barrier (BBB) ... 8
1.3 MRI-guided Laser Ablation (MLA) ... 10
1.4 Doxorubicin ... 11
1.5 DSC-MRI ... 12
1.6 Biomarkers of BBB Disruption ... 13
1.7 MLA and Immune Activation ... 14
1.8 Study Rationale ... 14
2.0 OBJECTIVES ... 15
2.1 Primary Objective ... 15
2.2 Secondary Objectives ... 15
2.3 Exploratory Objective ... 16
3.0 PATIENT SELECTION ... 16
3.1 Inclusion Criteria – Arm A ... 16
3.2 Inclusion Criteria – Arms B and C ... 16
3.3 Exclusion Criteria – Arm A ... 17
3.4 Exclusion Criteria – Arms B and C ... 17
3.5 Inclusion of Women and Minorities ... 18
4.0 REGISTRATION PROCEDURES ... 18
4.1 Confirmation of Patient Eligibility ... 19
4.2 Patient Registration in the Siteman Cancer Center Database ... 19
4.3 Assignment of UPN ... 19
4.4 Randomization ... 19
5.0 TREATMENT PLAN ... 20
5.1 Study Summary ... 20
5.2 MRI-Guided Laser Ablation ... 23
5.3 Premedication Administration ... 23
5.4 Agent Administration ... 23
5.5 Evaluability Criteria ... 24
5.6 General Concomitant Medication and Supportive Care Guidelines ... 24
5.7 Women of Childbearing Potential ... 24
5.8 Duration of Therapy ... 24
5.9 Duration of Follow-up ... 25
6.0 DOSE DELAYS/DOSE MODIFICATIONS ... 25
7.0 REGULATORY AND REPORTING REQUIREMENTS ... 28
7.1 Adverse Events (AEs) ... 28
7.2 Unanticipated Problems ... 28
7.3 Noncompliance ... 28
7.4 Serious Noncompliance ... 28
7.5 Protocol Exceptions ... 29
7.6 Reporting to the Human Research Protection Office (HRPO) and the Quality Assurance and Safety Monitoring Committee (QASMC) at Washington University ... 29

7.7 Timeframe for Reporting Required Events 29
8.0 PHARMACEUTICAL INFORMATION 30
8.1 Doxorubicin (Adriamycin) 30
9.0 CORRELATIVE STUDIES 32
9.1 DSC-MRI 32
9.2 Blood for Serum Biomarkers of BBB Disruption 32
9.3 Blood for Phenotyping of Peripheral Blood Immune Cells 33
9.4 Biopsy for Tumor-Specific Functional Activity of Peripheral Blood Immune Cells 34
9.5 Quality of Life Assessments 34
10.0 STUDY CALENDARS 35
10.1 Arm A Study Calendar 35
10.2 Arm B Study Calendar 36
10.3 Arm C Study Calendar 37
11.0 DATA SUBMISSION SCHEDULE 38
12.0 MEASUREMENT OF EFFECT 39
12.1 Antitumor Effect – Solid Tumors 39
12.2 Disease Parameters 41
12.3 Methods for Evaluation of Measurable Disease 42
13.0 DATA AND SAFETY MONITORING 44
14.0 STATISTICAL CONSIDERATIONS 45
15.0 REFERENCE 47
APPENDIX 1: Karnofsky Performance Scale 51
APPENDIX 2: Mini-Mental Status Exam 52

1.0 BACKGROUND AND RATIONALE

1.1 Glioblastoma Multiforme (GBM)

Glioblastoma multiforme (GBM) is the most common and the deadliest malignant brain tumor in adults[1]. The current standard chemoradiotherapy for newly diagnosed GBM produces only a modest survival benefit, and almost all patients die of their disease within five years [2,3]. For recurrent GBM, few effective treatments are available. Surgery is rarely an option in recurrent GBM. Bevacizumab, an anti-VEGFA monoclonal antibody, is currently the only approved therapy for recurrent GBM. However, its effect is temporary as it is thought to induce or select for highly resistant cancer cells [4-6]. It is clear that novel treatment approaches are needed to improve long-term survival in GBM patients.

1.2 Penetrating the Blood Brain Barrier (BBB)

Several cytotoxic and targeted agents have been tested in GBM patients with minimal success[7], despite the fact that these agents have shown significant anti-growth activity in cultured GBM cells. The high failure rate is in parts due to the redundancy in key growth pathways and in drug resistance of GBM cells. Another explanation is the poor CNS penetration many of these drugs have due to the blood brain barrier (BBB). As a result, high doses of drugs were used in these studies to achieve therapeutic drug concentrations in the CNS, which led to significant systemic toxicities and limited their clinical usefulness [7]. Thus, an outstanding challenge in neuro-oncology has been to generate drugs that have excellent CNS penetration or methods that can compromise the BBB to enhance drug delivery.

GBM typically appear on MR imaging as rim-enhancing masses, suggesting that the BBB within the growth-intensive rim is impaired because contrast enhancement reflects increased BBB permeability [8,9]. Consequently, delivery of cytotoxic agents to the tumor rim is higher than that to normal brain tissue. However, beyond the enhancing rim – the peritumoral region – where most micrometastatic GBM cells reside, the BBB remains relatively intact as demonstrated by the lack of contrast enhancement. As a result, access of cytotoxic drugs to this region is predicted to be more limited. Similar findings have also been observed in brain metastases arising from extracranial tumors. In many cancers, responses of brain metastases to systemic chemotherapy tend to closely parallel those of extra-cranial tumors [10-15]. Yet brain metastasis still represents a significantly poorer prognostic indicator than extracranial metastasis. This is likely because of the inherent nature of brain metastatic foci compromising critical neurological functions. Another possible explanation for this apparent paradox is that although cytotoxic agents can readily access brain metastases (BBB disrupted), they fail to reach therapeutic levels in the peritumoral or distant foci of brain micrometastases (BBB intact). Evidence supporting this theory came from studies in which drug levels of several cytotoxic agents were sampled in tumors and the surrounding normal brain tissue at the time of surgery or autopsy. Drug concentrations were at the highest in the tumors, and then rapidly decreased up to 40 fold lower within 2 cm distance from the viable tumor edge [16-18].

Overall, these results support two notions: 1) the BBB and its integrity negatively correlate with delivery and therapeutic effect of cytotoxic agents; and 2) if we can disrupt the BBB in the peritumoral region, we could improve cytotoxic chemotherapy delivery in this area. In GBM, most recurrent tumors arise within the peritumoral region – in one series, 90% of GBM relapses occurred within 2-3 cm margin of the primary site [19] - therefore elimination of micrometastatic GBM cells in this area will likely improve long-term disease control.

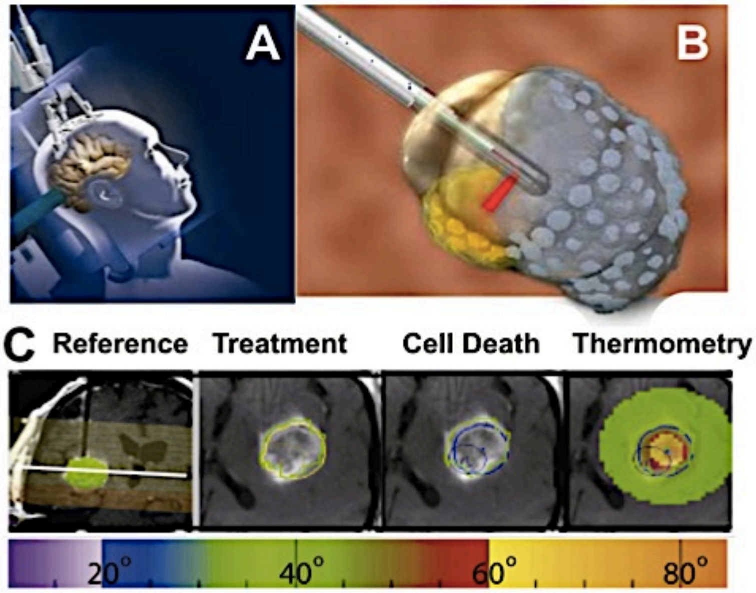

Figure 1: MRI-guided Heat Ablation Therapy (Monteris AutoLITT™). (A) Tripod base affixed to the skull with stereotactic wand visible. (B) Rendering of laser heating tumor from inside out. (C) Targeting strategy, treatment and cell death areas, and thermometry measurements. The target region was defines preoperatively based on MRI images (green sphere and green lines). Treatment areas were defines intraoperatively for each trajectory (yellow lines showing treatment area for trajectory #2). Cell death margins was determined as defined by trajectory and thermometry: thin and thick blue lines indicate cell death margins for trajectories #1 and #2, respectively. Thermometry measurements in degrees Celsius for the total treatment are plotted relative to the MRI.

To circumvent the BBB problem in local drug delivery, recent approaches have focused on bypassing it. A common method is the use of Gliadel wafers, a polymer implant impregnated with the chemotherapeutic agent BCNU and placed intraoperatively in the resection cavity to evade the BBB. This approach resulted in a statistically significant but modest survival advantage in both newly diagnosed and recurrent GBM [20-22]. The modest benefit of Gliadel could be due to the short duration of drug delivery – most BCNU is released over a period of 5 days[23]. However, the fact that direct delivery of a cytotoxic drug into the resection cavity for as little as 5 days could improve survival of GBM patients to a degree approaching that achieved by 8 months of systemic temozolomide chemotherapy is remarkable in itself, supporting the theory that the BBB is critical to cytotoxic chemotherapy effect. Unfortunately, Gliadel is not widely utilized as it requires a major surgery and can impair wound healing. Another approach of bypassing the BBB is the convection enhanced delivery system, in which a catheter is surgically inserted into the tumor to deliver chemotherapy [24]. This invasive procedure requires prolonged hospitalization, meticulous maintenance of the external catheter to prevent serious complications, and as a result remains investigational and is rarely used.

1.3 MRI-guided Laser Ablation (MLA)

MLA is a minimally invasive laser surgery currently FDA approved for cytoreductive treatment of brain tumors, both primary and metastatic [25]. Dr. Eric Leuthardt at Washington University performed one of the first MLA procedures in the country and is an international expert in laser ablation treatment of brain tumors. Currently Washington University has one of the largest clinical experiences using this new technology (Monteris, Winnipeg, CA). MLA employs a small incision in the scalp and skull, through which a thin laser probe is inserted and guided by MR imaging to the core of a tumor mass where it delivers hyperthermic ablation from the core to the rim. The maximal temperature in the core can reach greater than 70°C resulting in coagulative necrosis (Fig. 1). The temperature decreases in the peritumoral region but remains high enough (>40°C) to induce changes in the BBB as evidenced by new peritumoral contrast enhancement extending several centimeters from the tumor edge, while the original tumor enhancement is lost due to the heat ablation (Figs. 1 & 2). These observations suggest that an interesting side effect of MLA is the disruption of the peritumoral BBB. These changes often persist for several weeks (not shown), providing a rare window of opportunity during which drug delivery can be enhanced to eliminate infiltrative tumor cells residing in this region where most recurrences occur.

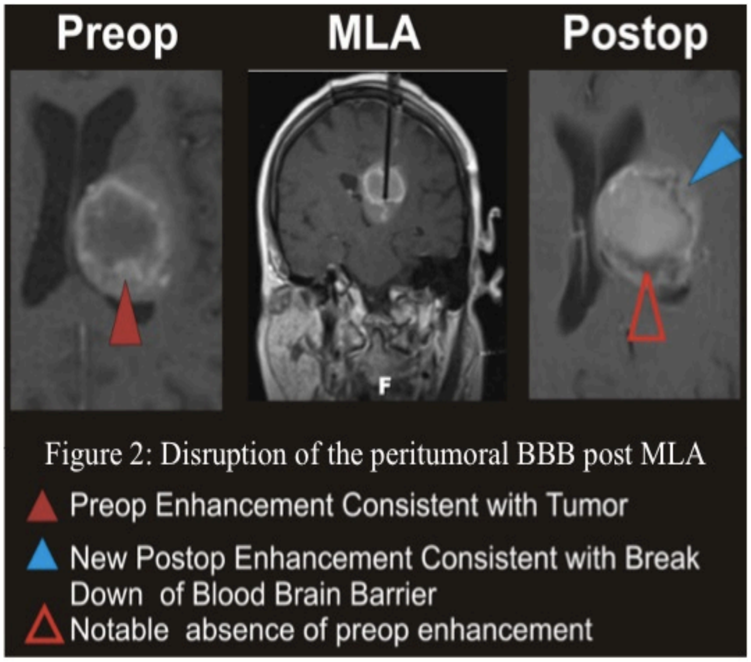

Figure 2: Disruption of the peritumoral BBB post MLA
▲ Preop Enhancement Consistent with Tumor
▲ New Postop Enhancement Consistent with Break Down of Blood Brain Barrier
△ Notable absence of preop enhancement

Whether MLA indeed causes BBB disruption remains unexplored and is the main focus of this study. Interestingly, the role of hyperthermia in inducing increased BBB permeability has been previously described in several animal models. In a rodent model of human glioma, the global heating of a mouse's head to 42°C for 30 minutes in a warm water bath significantly increased the maximal brain concentration of a thermosensitive liposome encapsulated with the chemotherapeutic drug Adriamycin [26]. To effect locoregional hyperthermia in the brain, retrograde infusion of a hyperthermic saline solution at 43°C into the left external carotid artery in the Wistar rat model reversibly increased BBB permeability to Evans-blue albumin in the left cerebral hemisphere[27]. In the most analogous method to the MLA, Nd:YAG laser-induced thermo-therapy to the left forebrain of Fischer rats resulted in locoregional disruption of the BBB as evidenced by increased locoregional passage of the Evans blue dye, serum proteins (e.g. fibrinogen

and IgM), and the cytotoxic drug paclitaxel [28]. Based on these results, we hypothesize that hyperthermia-induced disruption of the peritumoral BBB by MLA (Fig. 2) represents a potentially powerful tool to enhance delivery of chemotherapy to this region to effectively target residual disease in addition to maximal cytoreduction of the tumor.

1.4 Doxorubicin

The ideal cytotoxic drug for the purpose of this proposal should be one that has potent activities against GBM cell lines *in vitro*, yet has limited clinical utility in GBM treatment due to its poor BBB permeability, and that becomes effective against GBM *in vivo* when it gains access to the brain parenchyma. Of the most commonly used cytotoxic agents, doxorubicin is the best candidate. Doxorubicin has been shown to kill a large number of high grade glioma cell lines *in vitro*[29]. However, it has poor CNS penetration and has not been used extensively in CNS tumors. Doxorubicin and other anthracyclines induce cytotoxicity through intercalating between DNA base pairs, thereby interfering with strand elongation by DNA and RNA polymerase. Doxorubicin also affects topoisomerase II, which creates temporary double-strand DNA breaks during DNA replication. Doxorubicin stabilizes the DNA-topoII complex leading to double-strand DNA breaks and cell death.

Doxorubicin has a wide volume of distribution with tissue levels proportional to the DNA content of the tissue. Doxorubicin is 75% bound to plasma proteins. Doxorubicin is mostly metabolized in the liver and eliminated mainly as glucuronide or hydroxylated conjugates in the bile and feces. The half-life of doxorubicin is 1 to 3 hours.

In this study, we are testing the concept that increased delivery of cytotoxic chemotherapy to the peritumoral region after MLA will result in increased peritumoral disease control. Although this approach appears to be similar to the dose escalation method, one clear difference is that in the dose escalation method, increasing systemic doses of drugs are used to achieve adequate drug concentrations in the CNS at the expense of significant systemic and global CNS toxicities, especially at doses near or exceeding the maximal tolerated dose. Therefore, the lack of benefits at high systemic doses may in part be due to excessive toxicities. On the other hand, in the MLA-enhanced drug delivery system, lower doses of drugs will be given more frequently to limit systemic toxicities and to selectively concentrate drugs in the peritumoral region where therapeutic action is desired, thus also reducing CNS toxicities.

In solid tumors, doxorubicin is usually given in combination with other cytotoxic drugs. In breast cancer, doxorubicin is given at 50-60 mg/m^2 IV every 3 weeks in combination with cyclophosphamide. In bladder cancer, doxorubicin is given at 30 mg/m^2 IV every 4 weeks in combination with cisplatin, vinblastine, and methotrexate. Since we will use doxorubicin alone, we can give it at a lower dose and more frequently yet still achieve the same total dose as in other cancers such as breast cancer. This is to minimize systemic toxicity while still achieving adequate drug delivery in the peritumoral region if the BBB is disrupted. Therefore, we will use doxorubicin at 20 mg/m^2 IV every week during the window of MLA-induced peritumoral BBB disruption (6 weeks).

1.5 DSC-MRI

The measurement of perfusion in the brain using MRI is now commonplace and traces its origin to the seminal paper by Ostergaard et al.[30]. This technique is now referred to as dynamic susceptibility contrast (DSC) and relies on measuring the T2* signal changes that occur in the brain as a bolus of contrast material dynamically passes through the capillary circulation. DSC methods have since proved valuable in the diagnosis of stroke and brain tumors and are routinely used in our radiology practice. The mathematical model that describes DSC assumes that the blood brain barrier (BBB) is intact, but this assumption does not often hold in enhancing high-grade glial tumors, which are often supplied by leaky neo-vascularity. The leakage of contrast into the extracellular space causes changes in the T1 signal[31], which can invalidate uncorrected DSC measurements in tumors. Several methods have been proposed to correct for this leakage[31,32], some of which rely on a 2-compartment pharmacokinetic model that can estimate the vascular transfer constant (Ktrans)[32]. Ktrans is a parameter that describes the ability of contrast to move from the intravascular compartment to the extracellular compartment (the 2

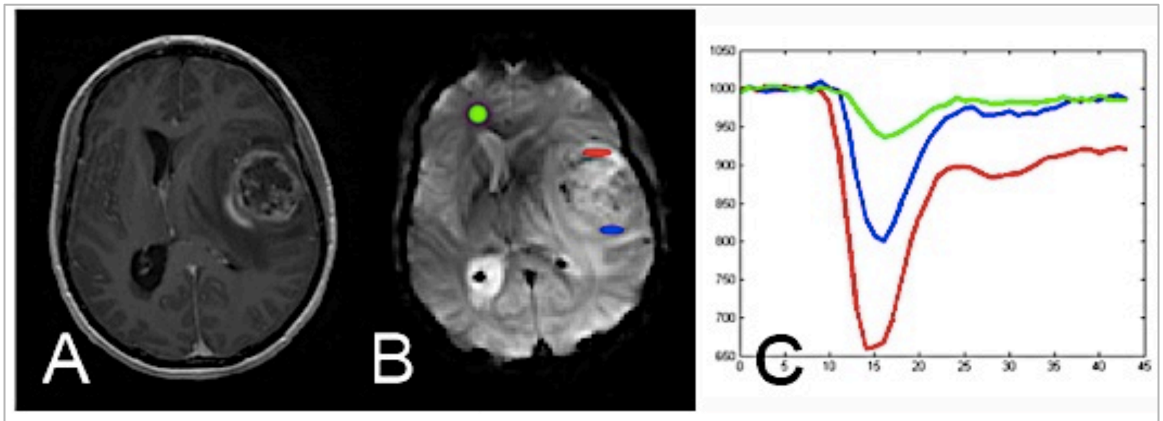

Figure 3: Perfusion Measurements in patient with GBM. See text for details.

compartments in the model) and thus provides a quantitative measure of the degree of BBB leakage. Law et al. used this model to measure Ktrans and cerebral blood volume (CBV) in 74 patients with glial tumors and demonstrated that the combination of both measures provided the best discriminator for high-grade gliomas, thus providing validation for this model[33].

We implemented a modified version of the Johnson model[32] and applied it to several patients with high-grade gliomas as illustrated in Figure 3. Figure 3A is a T1-weighted post contrast image of the tumor, seen as a ring enhancing mass on the left side of the brain (right side of the image). Figure 3B is the same slice in a T2*-weighted image that is used to measure the signal change with the passage of a bolus of contrast material. Several regions of interest (ROI) are drawn on this image where measurements were made. The red ROI was placed in an area of relatively large BBB leakage, the blue ROI in an area of less leakage, and the green ROI in normal white matter. Figure 3C demonstrates the normalized tissue signal curves during the bolus tracking period that

correspond to the drawn ROIs. The large signal drop seen centered at time frame 15 is taken at the peak of the contrast bolus transition. The depth and recovery of the signal during and after the bolus passage provides information on the degree of BBB leakage and on the value of Ktrans. In the normal white matter (green) the signal nearly recovers fully to its original value, consistent with the residual contrast left in the blood stream after the passage of the primary bolus. In the enhancing portion of the tumor (red and blue) the signal has a larger drop and does not fully recover providing us with estimates of the Ktrans. The measured Ktrans values in the most leaky portion of the tumor was estimated at Ktrans (red) = 0.21 min^{-1}; and in the intermediate area at Ktrans (blue) = 0.14 min^{-1}, which compares well with values given in Law et al.[33] of Ktrans = 0.29 min^{-1}.

We will employ a similar technique to determine the degree, extent, and duration of BBB leakage in the peritumoral region after MLA procedure. This will generate a dynamic and temporal map of BBB disruption in the area surrounding the post MLA tumor. Our long-term goal is to correlate these maps with patients' treatment outcome, when we have a larger database of treated patients, to determine whether patients with larger degree and extent of BBB leakage also derive more benefit from early chemotherapy. We may need to increase imaging frequency from every 2 weeks to weekly if post MLA BBB leakage is more transient than anticipated. In the event that DSC-MRI fails to produce a reliable measurement of BBB leakage, an alternative approach is high-resolution anatomical T1- and T2-weighted, dynamic contrast-enhanced MRI (DCE-MRI). DCE-MRI is another common method used to evaluate for permeability of blood vessels in the brain[9]. Similar to DSE-MRI, DCE-MRI consists of injection of an MRI contrast agent followed by multiple T1-weighted images to assess the leakage of this agent into the extracellular space over several minutes. Again, the rate of pooling of the contrast agent is then used to quantify the degree of BBB permeability.

1.6 Biomarkers of BBB Disruption

The coagulative necrosis and BBB disruption induced by MLA share several parallels with classical brain injuries (e.g. traumatic, surgical, ischemic, or pathologic brain damage), albeit in a more controlled setting. Several serum biomarkers have been identified, and in some cases extensively validated, in large number of patients with various forms of brain injuries. The compromised BBB after brain injuries allows CNS-specific factors released by damaged CNS cells to escape into the peripheral circulation where they can be detected using highly sensitive and specific detection techniques such as ultrasensitive ELISA, antibody arrays and HPLC/mass spectroscopy. The temporal profile of serum levels of these brain-specific factors (e.g. S100B, GFAP, brain-specific enolase or BSE, the brain-specific microRNA 124 or miR-124) can provide information about the duration and degree of BBB disruption[34-44], irrespective of the type of brain injuries.

We will measure serum levels of these 4 brain-specific factors (S100B, GFAP, BSE, and miR-124) immediately before and after MLA and then weekly for 6 weeks, using ultrasensitive ELISA (S100B, GFAP, BSE) or qPCR (miR-124). If MLA results in sustained BBB disruption, we expect that the level of at least one of these biomarkers will

increase precipitously soon after MLA and well above that caused by recurrent GBM, and that the increase will persist for several days to weeks. Antibody microarray can substitute ELISA if higher detection sensitivity is needed. We chose these biomarkers as they have previously been validated in brain trauma, recognizing that other brain specific factors may be more reliable. Also to confirm that the increased serum levels of these factors were due to the immediate and delayed thermal effect of MLA and not merely the result of trauma caused by the MLA probe, we will compare the serum levels of the above 4 factors as well as of the proteasomes 20S/26S complex, which has been implicated in tissue burn injuries[45], in post-MLA patients to those of 5 control patients who receive a needle biopsy of their brain tumors for diagnostic purposes without heating.

1.7 MLA and Immune Activation

One of the most striking findings from the Washington University experience with MLA is the delayed timing, degree, and persistence of peritumoral enhancement following treatment. Specifically, strong enhancement is observed several days after treatment and persists beyond 6 weeks. We hypothesize that these imaging findings are due to persistent disruption of the BBB, which is compounded and maintained by an enhanced immune infiltrate in the peritumoral area. Clinically, we have observed improved patient outcomes in our retrospective MLA series (Table 1) and hypothesize that this is in part due to an MLA-induced anti-tumor immune response. CNS immunosurveillance is likely distinct when compared to other tissues with clearly defined secondary lymphoid structures[46]. However, CNS antigen presentation is thought to occur when antigens drain to the ipsilateral cervical lymph node chain[46]. Thus, it is possible that MLA disrupts the BBB such that tumor antigens, either native or heat denatured, have greater access to cervical draining lymphoid tissue, thereby stimulating an anti-glioma immune response that (a) prolongs patient survival and (b) leads to persistent contrast enhancement characteristic of this treatment.

1.8 Study Rationale

By employing a combination of advanced MRI techniques and correlative serum biomarkers of BBB disruption, we plan to develop a powerful, first of its kind clinical algorithm whereby we can measure and identify the window of maximal BBB disruption post MLA to 1) allow for optimal chemotherapeutic dosing to achieve the greatest benefits and the least systemic side effects and 2) distinguish subsequent tumor progression from long-term MLA treatment effects. This pilot therapeutic study will provide preliminary validation of the algorithm.

Although we have a large armamentarium of cytotoxic drugs with potential activity against GBM, the vast majority has poor CNS penetration limiting their usefulness in GBM treatment. In addition, the frequent use of bevacizumab in GBM treatment further diminishes CNS uptake of cytotoxic drugs as it decreases BBB permeability[6,47,48]. Our proposed use of MLA to achieve both cytoreduction and increased permeability of the peritumoral BBB has the potential to be practice changing and will allow us to test many

drugs that have not shown promise in GBM therapy due to poor BBB penetration. The innovative algorithm to detect and measure MLA-induced peritumoral BBB compromise may also be applied to treatments of other primary brain tumors as well as brain metastases.

Recent work has collectively demonstrated striking immune dysregulation in patients with GBM, including T cell lymphopenia and anergy, cytokine dysregulation, and increased regulatory T cell (T_{reg}) populations among others, which reflect immunologic compromise and functional impairment[46,49-51]. However, a growing list of potential tumor antigens has been identified, suggesting that tumor-specific recognition by immune cells may be biologically relevant and therapeutically exploitable[50,52]. Therefore, given the immune dysfunction characterized in GBM patients, approaches that potentiate the anti-glioma immune response are particularly exciting. A priori, because the immune system has evolved to recognize a tremendous diversity of antigenic epitopes in vertebrates, immune-potentiating efforts – with MLA being one potential approach – may be especially effective at targeting the heterogeneity that defines GBM.

2.0 OBJECTIVES

2.1 Primary Objective

1. To determine MR imaging correlates of peritumoral BBB disruption after MLA.

2. To identify serum biomarkers of peritumoral BBB disruption after MLA, which can be used to establish peritumoral permeability scores.

3. To determine 6-month progression-free survival (6PFS) in patients who receive doxorubicin immediately following MLA and patients who receive doxorubicin beginning 6-8 weeks after MLA as compared to the historical control of bevacizumab alone.

2.2 Secondary Objectives

1. To determine the predictive value of the peritumoral permeability score for patient outcome as measured by 6-PFS rate.

2. To determine overall survival in patients who receive doxorubicin immediately following MLA and patients who receive doxorubicin beginning 6-8 weeks after MLA as compared to the historical control of bevacizumab alone.

3. To evaluate quality of life (QOL) using Karnofsky performance status and the mini-mental state examination (MMSE, see Appendix 2) in patients who receive doxorubicin immediately following MLA and patients who receive doxorubicin beginning 6-8 weeks after MLA.

2.3 Exploratory Objective

1. To investigate the correlation between the duration of MLA-induced BBB disruption (as determined by MRI correlates and biomarkers) and 6-month PFS.

2. To determine the effects of MLA treatment on patient's tumor-specific immune responses.

3.0 PATIENT SELECTION

3.1 Inclusion Criteria – Arm A

1. Newly diagnosed brain tumors that appear high-grade based on MRI.

2. Scheduled for biopsy for diagnostic purposes.

3. At least 18 years of age.

4. Ability to understand and willingness to sign an IRB-approved written informed consent document.

3.2 Inclusion Criteria – Arms B and C

1. Histologically confirmed GBM; rare GBM variants, secondary GBM, and suspected secondary GBM are allowed.

2. Unequivocal evidence of tumor progression by MRI scan (see Section 12).

3. There must be an interval of at least 12 weeks from the completion of radiotherapy to study registration except if there is unequivocal evidence for tumor recurrence per RANO criteria (see Section 12). When the interval is less than 12 weeks from the completion of radiotherapy, the use of PET scan is allowed to differentiate between unequivocal evidence of tumor recurrence and pseudoprogression.

4. Largest dimension of the recurrent tumor is a maximum of approximately 3 cm (the maximal tumor size for optimal MLA).

5. At least 18 years of age.

6. Karnofsky performance status ≥ 60%, see Appendix 1.

7. Scheduled for MLA.

8. Normal left ventricular ejection fraction on MUGA or echocardiogram within the past 1 year prior to registration for patients with history of congestive heart failure and/or

coronary disease requiring medications other than aspirin, or known prior exposure to anthracycline chemotherapy.

9. Adequate bone marrow and hepatic function as defined below (must be within 7 days of MLA):
   a. Absolute neutrophil count (ANC) ≥ 1500/mcl (G-CSF is allowed)
   b. Platelets ≥ 100,000/mcl
   c. Hemoglobin ≥ 9 (pRBC transfusion +/- ESA are allowed)
   d. ALT ≤ 3 x ULN
   e. AST ≤ 3 x ULN
   f. ALP ≤ 3 x ULN. If ALP is > 3 x ULN, GGT must be checked and be ≤ 3 x ULN.
   g. Bilirubin ≤ 2 x ULN

10. At the time of registration, patient must have recovered from the toxic effects of prior therapy to no more than grade 1 toxicity.

11. At the time of registration, patient must be at least 2 weeks from prior vincristine, 3 weeks from prior procarbazine, and 4 weeks from other prior cytotoxic chemotherapy.

12. Women of childbearing potential and men must agree to use adequate contraception (hormonal or barrier method of birth control, abstinence) prior to study entry and for the duration of study participation. Should a woman become pregnant or suspect she is pregnant while participating in this study, she must inform her treating physician immediately.

13. Ability to understand and willingness to sign an IRB approved written informed consent document (or that of legally authorized representative, if applicable).

3.3 Exclusion Criteria – Arm A

1. Currently receiving or scheduled to receive any other therapies intended to treat the newly diagnosed high-grade glioma prior to the biopsy and the post-biopsy biomarker sample collection and immune monitoring sample collection.

2. Pregnant.

3. Known history of HIV or autoimmune diseases requiring immunosuppressant drugs.

3.4 Exclusion Criteria – Arms B and C

1. Prior treatment with doxorubicin and/or bevacizumab.

2. Prior treatment with Gliadel wafer is allowed if it has been at least 3 months from placement.

3. Previous treatment with complete cumulative doses of daunorubicin, idarubicin, and/or other anthracyclines and anthracenediones that is equivalent to a total dose of 240 mg/m^2 doxorubicin.

4. More than 2 prior relapses.

5. Currently receiving any other investigational agents that are intended as treatments of GBM.

6. A history of allergic reactions attributed to compounds of similar chemical or biologic composition to doxorubicin or other agents used in the study.

7. Uncontrolled intercurrent illness including, but not limited to, ongoing or active infection, symptomatic congestive heart failure, unstable angina pectoris, cardiac arrhythmia, recent heart attack within the previous 12 months or severe heart problems, or psychiatric illness/social situations that would limit compliance with study requirements.

8. Pregnant and/or breastfeeding. Premenopausal women must have a negative serum or urine pregnancy test within 14 days of study entry.

9. Inability to undergo MRI due to personal and medical reasons.

10. Known history of HIV or autoimmune diseases requiring immunosuppressant drugs

3.5 Inclusion of Women and Minorities

Both men and women and members of all races and ethnic groups are eligible for this trial.

4.0 REGISTRATION PROCEDURES

Patients must not start any protocol intervention prior to registration through the Siteman Cancer Center.

The following steps must be taken before registering patients to this study:

1. Patients will provide consent to participate in trial
2. Confirmation of patient eligibility
3. Registration of patient in the Siteman Cancer Center database
4. Assignment of unique patient number (UPN)

4.1 Confirmation of Patient Eligibility

Confirm patient eligibility by collecting the information listed below:

1. The registering MD's name
2. Patient's race, sex, and DOB
3. Three letters (or two letters and a dash) for the patient's initials
4. Copy of signed consent form
5. Completed eligibility checklist, signed and dated by a member of the study team
6. Copy of appropriate source documentation confirming patient eligibility

4.2 Patient Registration in the Siteman Cancer Center Database

All patients must be registered through the Siteman Cancer Center database. Registration in the SCC database will be the last step of the patient registration process. Once the patient has been registered in the SCC database, s/he will be considered registered to the study.

4.3 Assignment of UPN

Each patient will be identified with a unique patient number (UPN) for this study. UPNs for this study start at 1000 and will be assigned in sequential order as patients are enrolled. Patients will also be identified by first, middle, and last initials. If the patient has no middle initial, a dash will be used on the case report forms (CRFs). All data will be recorded with this identification number on the appropriate CRFs.

4.4 Randomization

After 10 patients are enrolled in Arm B, the remaining 30 patients will be randomized in a 2:1 ratio to receive either immediate doxorubicin following MLA (Arm C) or late doxorubicin following MLA (Arm B). Randomization will be performed by a centralized computer randomization process and will take place at the same time as registration.. Registration and randomization must be performed before MLA and prior to obtaining the second biomarker lab sample and the second DSC-MRI (approximately 72 hours after MLA). If a patient with suspected secondary GBM is randomized to Arm C and the pathology results are not available within 7 days of the MLA procedure or by the time the patient is scheduled to start treatment with doxorubicin, s/he will not be treated and will be removed from study. "Determined to be ineligible" will be listed as the "Reason Off Therapy" in the SCC database. The date pathology results were reviewed and/or the date the final determination about eligibility was made will be used as the "Date Off Therapy." Patients removed from study due to suspected secondary GBM not being confirmed through pathology will not be included in the 10 patient total enrolled in Arm B or the remaining 30 patients randomized to Arms B or C and will be replaced.

5.0 TREATMENT PLAN

5.1 Study Summary

5.1.1 Arm A

Five patients enrolled to this study will be in Arm A; patients may be enrolled to Arm A at any time throughout the lifetime of the study. Arm A patients will undergo biopsy of their primary brain tumors (newly diagnosed or recurrent) for standard of care diagnostic purposes but will not undergo MLA and will not receive doxorubicin.

These patients will also have 5 mL blood for serum and 2 mL blood for plasma drawn at the following time points:

- before biopsy; this sample can be collected any time during the 3 days before or the day of the procedure until the start of the procedure
- within approximately 3 days after biopsy
- 2 weeks (+/- 3 days) after biopsy
- 4 weeks (+/- 3 days) after biopsy

The purpose of these blood draws is to measure serum levels of brain-specific factors (such as S100B, GFAP, and BSE) (see Section 9.2.)

Arm A patients will also have blood drawn at the following time points:

- before biopsy; this sample can be collected any time during the 3 days before or the day of the procedure until the start of the procedure (40 mL of blood)
- 2 weeks (+/- 3 days) after biopsy (20 mL of blood)
- 4 weeks (+/- 3 days) after biopsy (20 mL of blood)
- every 2 weeks (+/- 3 days) thereafter for up to 3 months after biopsy provided there is no evidence of significant chemotherapy-induced leukopenia (defined as leukopenia and/or neutropenia that required growth factor support or persistent lymphopenia (ALC < 500 or CD4 < 200 for > 2 months) that requires chemoprophylaxis for opportunistic infections) (20 mL of blood). If leukocyte counts (WBC) are not adequate, the blood draw will be skipped at that particular time point. If the leukocyte counts remain inadequate for more than 4 weeks, the blood draw will be discontinued and the patient will cease to participate in the study.

The purpose of these blood draws is to isolate dendritic cells (for the pre-biopsy sample) and PBMC (for the pre- and post-biopsy samples) to determine the phenotypes and functions of peripheral blood immune cells following biopsy (see Section 9.3).

Patients in Arm A will not start definitive therapy for their brain tumor until at least 4 weeks from the date of biopsy.

Arm A patients will have one follow-up visit within 7 to 14 days after the final blood samples are obtained to determine if the patient experienced any reportable adverse events following the final blood draw. These patients will then cease to participate in this study. No further data, short-term, or long-term follow-up of these patients will be required for this study.

5.1.2 Arm B

Arm B patients will undergo MLA and will begin doxorubicin (as described in Section 5.4) 6 to 8 weeks following MLA. Please see Section 4.4 for information on randomization to Arms B and C following enrollment of the first 10 participants to Arm B.

Arm B patients will undergo DSC-MRI at the following time points:

- no more than 2 weeks prior to MLA
- within approximately 3 days after MLA
- 2 weeks (+/- 3 days) after MLA
- 4 weeks (+/- 3 days) after MLA
- 6 weeks (+/- 3 days) after MLA
- 10 weeks (+/- 7 days) after MLA **only if** the 6-week scan shows prolonged disruption of the blood brain barrier as evidenced by persistent contrast enhancement.
- 14 weeks (+/- 7 days) after MLA and then every 8 weeks (+/- 7 days) thereafter until disease progression (see Section 9.1)

Arm B patients will have 5 mL blood for serum and 2 mL blood for plasma drawn at the following time points:

- Before MLA; this sample can be collected any time during the 3 days before or the day of the procedure until the start of the procedure
- Within approximately 3 days after MLA
- 1 week (+/- 3 days) after MLA
- 2 weeks (+/- 3 days) after MLA
- 3 weeks (+/- 3 days) after MLA
- 4 weeks (+/- 3 days) after MLA
- 5 weeks (+/- 3 days) after MLA
- 6 weeks (+/- 3 days) after MLA
- 10 weeks (+/- 7 days) after MLA **only if** the 6-week post-MLA scan shows prolonged disruption of the blood brain barrier as evidenced by persistent contrast enhancement.
- 14 weeks (+/- 7 days) after MLA and then every 8 weeks (+/- 7 days) until disease progression

The purpose of these blood draws is to measure serum levels of brain-specific factors (such as S100B, GFAP, and BSE) (see Section 9.2.). This is in addition to

blood samples for routine laboratory prior to chemotherapy and surgery.

Finally, the second 10 patients enrolled to Arm B (those who are randomized to Arm B, not the first 10 patients enrolled to Arm B without being randomized) will undergo a biopsy at the time of MLA to collect tumor tissue for the immune analysis if archival tissue is not available (see Section 9.4).

For these second 10 patients enrolled to Arm B, blood samples will be drawn at the following time points:

- Before biopsy and MLA; this sample can be collected any time during the 3 days before or the day of the procedure until the start of the procedure (40 mL of blood)
- 2 weeks (+/- 3 days) after biopsy and MLA (20 mL of blood)
- 4 weeks (+/- 3 days) after biopsy and MLA (20 mL of blood)
- Every 2 weeks (+/- 3 days) thereafter for up to 3 months after biopsy and MLA provided there is no evidence of significant chemotherapy-induced leukopenia (defined as leukopenia and/or neutropenia that required growth factor support or persistent lymphopenia (ALC < 500 or CD4 < 200 for > 2 months) that requires chemoprophylaxis for opportunistic infections) (20 mL of blood). If leukocyte counts (WBC) are not adequate, the blood draw will be skipped at that particular time point. If the leukocyte counts remain inadequate for more than 4 weeks, the blood draw will be discontinued.

The purpose of these blood draws is to isolate dendritic cells (for the pre-biopsy and MLA sample) and PBMC (for the pre- and post-biopsy and MLA samples) to determine the phenotypes and functions of peripheral blood immune cells following biopsy (see Section 9.3).

5.1.3 Arm C

Arm C patients will undergo MLA and will begin doxorubicin (as described in Section 5.4) within approximately 7 days following MLA.

Arm C patients will undergo DSC-MRI at the following time points:

- no more than 2 weeks prior to MLA
- within approximately 3 days after MLA
- 2 weeks (+/- 3 days) after MLA
- 4 weeks (+/- 3 days) after MLA
- 6 weeks (+/- 3 days) after MLA
- 10 weeks (+/- 7 days) after MLA **<u>only if</u>** the 6-week scan shows prolonged disruption of the blood brain barrier
- 14 weeks (+/- 7 days) after MLA and then every 8 weeks (+/- 7 days) until disease progression (see Section 9.1)

Arm C patients will have 5 mL blood for serum and 2 mL blood for plasma drawn at the following time points:

- before MLA; this sample can be collected any time during the 3 days before or the day of the procedure until the start of the procedure
- within approximately 3 days after MLA
- weekly (+/- 3 days) before (on the same day as) chemotherapy
- weekly (+/- 3 days) within 24 hours after chemotherapy
- 10 weeks (+/- 7 days) after MLA **only if** the 6-week post-MLA scan shows prolonged disruption of the blood brain barrier
- 14 weeks (+/- 7 days) after MLA and then every 8 weeks (+/- 7 days) until disease progression

The purpose of these blood draws is to measure serum levels of brain-specific factors (such as S100B, GFAP, and BSE) (see Section 9.2.). This is in addition to blood samples for routine laboratory prior to chemotherapy and surgery.

Please note that if the first doxorubicin dose in Arm C is given within 24 hours of the post-MLA biomarker lab, the post-MLA biomarker lab can be used as the pre-chemotherapy biomarker lab for this time point. During the weeks of DSC-MRI, biomarker lab must be drawn on the same day as DSC-MRI (prior to the scan).

5.2 MRI-Guided Laser Ablation

MLA is a minimally invasive laser surgery currently FDA approved for cytoreductive treatment of brain tumors, both primary and metastatic [25]. MLA employs a small incision in the scalp and skull, through which a thin laser probe is inserted and guided by MR imaging to the core of a tumor mass where it delivers hyperthermic ablation from the core to the rim. The maximal temperature in the core can reach greater than 70°C resulting in coagulative necrosis.

For patients with suspected secondary GBM, MLA should take place early in the week (no later than Tuesday) so that pathology confirming the diagnosis will be back in time for treatment should the participant be randomized to Arm C.

5.3 Premedication Administration

Because doxorubicin is emetogenic, prophylactic use of antiemetics will be as follows: palonosetron 0.25 mg IVP and dexamethasone 10 mg IV 30 minutes prior to each dose of doxorubicin.

5.4 Agent Administration

Doxorubicin will be given intravenously on an outpatient basis weekly for 6 weeks at a dose of 20 mg/m^2 over 5 minutes. Patients in Arm B will begin doxorubicin 6-8 weeks after MLA; patients in Arm C will begin doxorubicin within approximately 7 days after MLA.

5.5 Evaluability Criteria

All patients in Arms B and C are evaluable for toxicity. Patients are evaluated from first receiving study treatment until a 30-day follow up after the conclusion of treatment or death.

All patients in Arms B and C are evaluable for efficacy after receiving at least 2 doses of doxorubicin.

5.6 General Concomitant Medication and Supportive Care Guidelines

Subjects should not receive medications that may interact with doxorubicin. These include progesterone, verapamil, phenytoin, cyclosporine, phenobarbital, and streptozocin. Refer to the doxorubicin product label for details. Therapies excluded during the conduct of this trial include chemotherapy (other than doxorubicin), hormonal therapy for cancer, and other tumor-targeted therapies including but not limited to radiotherapy. Therapeutic use of hematopoietic colony-stimulating factors is permitted following ASCO guidelines.

5.7 Women of Childbearing Potential

Women of childbearing potential (defined as women with regular menses, women with amenorrhea, women with irregular cycles, women using a contraceptive method that precludes withdrawal bleeding, and women who have had a tubal ligation) are required to have a negative serum pregnancy test within 14 days prior to biopsy/MLA.

Female and male patients (along with their female partners) are required to use two forms of acceptable contraception, including one barrier method, during participation in the study and for 2 months following the last dose of doxorubicin.

If a patient is suspected to be pregnant, doxorubicin should be immediately discontinued. In addition a positive urine test must be confirmed by a serum pregnancy test. If it is confirmed that the patient is not pregnant, the patient may resume dosing.

If a female patient or female partner of a male patient becomes pregnant during therapy or within 2 months after the last dose of doxorubicin, the investigator must be notified in order to facilitate outcome follow-up.

5.8 Duration of Therapy

If at any time the constraints of this protocol are considered to be detrimental to the patient's health and/or the patient no longer wishes to continue protocol therapy, the protocol therapy should be discontinued and the reason(s) for discontinuation documented in the case report forms.

In the absence of treatment delays due to adverse events, treatment with doxorubicin may continue for 6 weeks or until one of the following criteria applies:

- Documented and confirmed disease progression
- Death
- Adverse event(s) that, in the judgment of the investigator, may cause severe or permanent harm or which rule out continuation of study drug
- General or specific changes in the patient's condition render the patient unacceptable for further treatment in the judgment of the investigator
- Suspected pregnancy
- Serious noncompliance with the study protocol
- Lost to follow-up
- Patient withdraws consent
- Investigator removes the patient from study
- The Siteman Cancer Center decides to close the study

Patients who prematurely discontinue treatment for any reason will be followed as indicated in the study calendar.

5.9 Duration of Follow-up

Patients in Arms B and C will be followed every 8 weeks after the 14-week post-MLA scan (not including the 10-week post-MLA scan that may or may not be performed depending on evidence of BBB disruption) until 2 years have elapsed since patient registration or until death, whichever occurs first. Follow-up consists of collection of Karnofsky performance status, DSC-MRI, and blood for biomarkers until disease progression (or 2 years of elapsed since patient registration or death, whichever occurs first). After documented disease progression, vital status and subsequent treatment for GBM will be obtained every 8 weeks for the remainder of the 2 years or until death, whichever occurs first. Patients removed from study for unacceptable adverse events that are possibly, probably, or definitely related to study procedures will be followed until resolution or stabilization of the adverse event. "Stabilization" is defined as remaining at a consistent CTCAE version 4.0 grade of the event for two consecutive assessments.

6.0 DOSE DELAYS/DOSE MODIFICATIONS

Doxorubicin will be used at a low dose (20mg/m^2 IV every week). Therefore grade 3 and 4 adverse events associated with doxorubicin are expected to be relatively low. The following algorithms are meant as guidelines only:

Dose Level	Doxorubicin IV
0	20 mg/m^2 IV every week
-1	15 mg/m^2 IV every week
-2	10mg/m^2 IV every week

Congestive heart failure: Given the low dose and short duration of doxorubicin treatment, we do not anticipate significant cardiac toxicity. However, any grade of cardiac toxicity that occurs during doxorubicin treatment will require immediate discontinuation of chemotherapy and initiation of appropriate cardiac treatment.

Anemia: Since pRBC transfusion and ESA are allowed, we do not expect treatment delays due to grade 1 and 2 anemia. For grade 3 and 4 anemia, doxorubicin will be held, anemia corrected with transfusion +/- ESA, and other causes (hemolysis, bleeding or sequestration) of anemia, if suspected, reasonably ruled out, before doxorubicin can be restarted at -1 dose level.

Other hematologic abnormalities:

**Baseline Permissible ANC**	**Subsequent ANC**	**Growth factor support, Treatment Delay and Follow-up lab**	**Dose Level based on rates of abnormal lab recovery**
ANC ≥ 1500/mcl	ANC ≥ 1500/mcl	None	0
	ANC < 1500/mcl but > 500/mcl	G-CSF 480 mcg SQ qday x up to 5 days. Check CBC daily. When ANC ≥ 1500/mcl, restart treatment.	0 if ANC recovers to ≥ 1500/mcl within 5 doses of G-CSF.
			-1 if ANC recovers to ≥ 1500/mcl after 5 doses of G-CSF.
			-2 if ANC takes > 14 days to becomes permissible.
	ANC < 500/mcl	G-CSF 480 mcg SQ qday x up to 5 days. Check CBC daily. When ANC ≥ 1500/mcl, restart treatment.	-1 if ANC recovers to ≥ 1500/mcl within 5 doses of G-CSF.
			-2 if ANC recovers to ≥ 1500/mcl after 5 doses of G-CSF.
			Discontinue treatment if ANC takes > 21 days to becomes permissible.

**Baseline Permissible Platelets**	**Subsequent Platelet count**	**Treatment Delay and Follow-up lab§**	**Dose Level based on rates of abnormal lab recovery**
Platelet ≥ 100,000/mcl	Plt ≥ 100,000/mcl	None	0
	Plt < 100,000/mcl but > 50000/mcl	Hold treatment. Check CBC twice a week. When plt ≥ 100,000/mcl, restart treatment.	0 if plt recovers to ≥ 100,000/mcl within 3 days.
			-1 if plt recovers to ≥ 100,000/mcl within 14 days.
			-2 if plt takes > 14 days to recovers to ≥ 100,000/mcl.
	Plt < 50,0000/mcl but ≥ 10,000	Hold treatment. Check CBC twice a week. When plt ≥ 100,000/mcl, restart treatment. Transfuse as needed to keep plt ≥ 20,000.	-1 if plt recovers to ≥ 100,000/mcl within 7 days.
			-2 if plt recovers to ≥ 100,000/mcl within 21 days.
			Discontinue treatment if plt takes > 21 days to recovers to ≥ 100,000/mcl.

	Plt ≤ 10,000/mcl	Hold treatment. Check CBC twice a week. Transfuse as needed to keep plt ≥ 20,000. When plt ≥ 100,000/mcl without transfusion, restart treatment.	- 2 if plt recovers to ≥ 100,000/mcl within 21 days.
			Discontinue treatment if plt takes > 21 days to recovers to ≥ 100,000/mcl.

§ All follow-up labs for dose modifications are +/- 3 days.

Hepatic abnormalities:

Baseline Permissible LFT (ALT, AST, ALP*) & bilirubin	Subsequent LFT & bilirubin	Treatment Delay and Follow-up lab §	Dose Level based on rates of abnormal lab recovery
LFT ≤ 3 x ULN<br><br>AND<br><br>Bilirubin ≤ 2 x ULN	LFT ≤ 3 x ULN AND bilirubin ≤ 2 x ULN	None	0
	Any LFT > 3 but ≤ 6 x ULN<br><br>AND/OR<br><br>Bilirubin > 2 but ≤ 3 x ULN	Delay for up to 7 days. Recheck lab at days 3 and 7 and restart treatment when the abnormal LFT becomes ≤ 3 x ULN AND bilirubin ≤ 2 x ULN.	0 if abnormal lab becomes permissible in day 3 lab.
			-1 if abnormal lab becomes permissible in day 7 lab.
			-2 if abnormal lab takes > 14 days to become permissible.
	Any LFT > 6 but ≤ 10 x ULN<br><br>AND/OR<br><br>Bilirubin > 3 but ≤ 5 x ULN	Delay for up to 14 days Recheck lab at days 7 and 14 and restart treatment when the abnormal LFT becomes ≤ 3 x ULN AND bilirubin ≤ 2 x ULN.	-1 if abnormal lab becomes permissible in day 7 lab.
			-2 if abnormal lab becomes permissible in day 14 lab.
			Discontinue treatment if abnormal lab takes > 21 days to becomes permissible.
	Any LFT >10 x ULN<br><br>AND/OR<br><br>Bilirubin > 5 x ULN	Discontinue treatment and monitor LFT until resolution. Imaging study as clinically indicated.	Discontinue treatment.

* If only ALP is outside the permissible range, GGT needs to be checked. If GGT is ≤ 3 x ULN, ALP level will not be used in this algorithm. In that case, GGT will be used instead. If GGT is > 3 x ULN, ALP will be used in this algorithm.

§ All follow-up labs for dose modifications are +/- 3 days.

7.0 REGULATORY AND REPORTING REQUIREMENTS

7.1 Adverse Events (AEs)

Definition: Any unfavorable medical occurrence in a human subject including any abnormal sign, symptom, or disease.

Grading: The descriptions and grading scales found in the revised NCI Common Terminology Criteria for Adverse Events (CTCAE) version 4.0 will be utilized for all toxicity reporting. A copy of the CTCAE version 4.0 can be downloaded from the CTEP website.

Attribution (relatedness), Expectedness, and Seriousness: The definitions for the terms listed that should be used are those provided by the Department of Health and Human Services' Office for Human Research Protections (OHRP). A copy of this guidance can be found on OHRP's website:
http://www.hhs.gov/ohrp/policy/advevntguid.html

7.2 Unanticipated Problems

Definition:

- unexpected (in terms of nature, severity, or frequency) given (a) the research procedures that are described in the protocol-related documents, such as the IRB-approved research protocol and informed consent document; and (b) the characteristics of the subject population being studied;
- related or possibly related to participation in the research ("possibly related" means there is a reasonable possibility that the incident, experience, or outcome may have been caused by the procedures involved in the research); and
- suggests that the research places subjects or others at a greater risk of harm (including physical, psychological, economic, or social harm) than was previously known or recognized.

7.3 Noncompliance

Definition: failure to follow any applicable regulation or institutional policies that govern human subjects research or failure to follow the determinations of the IRB. Noncompliance may occur due to lack of knowledge or due to deliberate choice to ignore regulations, institutional policies, or determinations of the IRB.

7.4 Serious Noncompliance

Definition: noncompliance that materially increases risks, that results in substantial harm to subjects or others, or that materially compromises the rights or welfare of participants.

7.5 Protocol Exceptions

Definition: A planned deviation from the approved protocol that are under the research team's control. Exceptions apply only to a single participant or a singular situation.

Pre-approval of all protocol exceptions must be obtained prior to the event.

7.6 Reporting to the Human Research Protection Office (HRPO) and the Quality Assurance and Safety Monitoring Committee (QASMC) at Washington University

The PI is required to promptly notify the IRB of the following events:

- Any unanticipated problems involving risks to participants or others which occur at WU, any BJH or SLCH institution, or that impacts participants or the conduct of the study.
- Noncompliance with federal regulations or the requirements or determinations of the IRB.
- Receipt of new information that may impact the willingness of participants to participate or continue participation in the research study.

These events must be reported to the IRB within **10 working days** of the occurrence of the event or notification to the PI of the event. The death of a research participant that qualifies as a reportable event should be reported within **1 working day** of the occurrence of the event or notification to the PI of the event.

7.7 Timeframe for Reporting Required Events

Reportable adverse events will be tracked for 30 days following the last day of doxorubicin for patients in Arms B and C and for 7 days following the final blood draw for patients in Arm A. For patients in Arm A, only adverse events considered possibly, probably, or definitely related to the blood draw(s) need be reported. Adverse events that are possibly, probably, or definitely related to study procedures will be followed until resolution or stabilization of the event. "Stabilization" is defined as remaining at a consistent CTCAE version 4.0 grade of the event for two consecutive assessments.

Deaths	
Any reportable death while on study or within 30 days of study	Immediately, within 24 hours, to PI and the IRB
Any reportable death while off study	Immediately, within 24 hours, to PI and the IRB
Adverse Events/Unanticipated Problems	
Any reportable adverse events as described in Sections 7.1 and 7.2 (other than death)	Immediately, within 24 hours to PI and within 10 working days to the IRB
All adverse events regardless of grade and attribution should be submitted cumulatively	Include in DSM report
Noncompliance and Serious Noncompliance	
All noncompliance and serious noncompliance as described in Sections 7.3 and 7.4	Immediately, within 24 hours, to PI and within 10 working days to the IRB

8.0 PHARMACEUTICAL INFORMATION

8.1 Doxorubicin (Adriamycin)

8.1.1 Doxorubicin Description

Doxorubicin is a cytotoxic anthracycline antibiotic isolated from cultures of *Streptomyces peucetius* var. *caesius*. Doxorubicin consists of a naphthacenequinone nucleus linked through a glycosidic bond at ring atom 7 to amino sugar, daunosamine. Chemically, doxorubicin hydrochloride is (8S,10S)-10-[(3-Amino-2,3,6-tirdeoxy-a-L-lyxo-hexopyranosyl)-oxy]-8-glycoloyl-7,8,9,10-tetrahydro-6,8,11-trihydroxy-1-methoxy-5,12-naphthacenedione hydrochloride.

Chemical name: $C_{27}H_{29}NO_{11}\cdot HCl$
Molecular weight: 579.99

8.1.2 Clinical Pharmacology

The cytotoxic effect of doxorubicin on malignant cells and its toxic effects on various organs are thought to be related to nucleotide base intercalation and cell membrane lipid binding activities of doxorubicin. Intercalation inhibits nucleotide replication and action of DNA and RNA polymerases. The interaction of doxorubicin with topisomerase II to form DNA-cleavable complexes appears to be an important mechanism of doxorubicin cytocidal activity.

8.1.3 Pharmacokinetics and Drug Metabolism

Pharmacokinetic studies, determined in patients with various types of tumors undergoing either single or multi-agent therapy, have shown that doxorubicin follows a mulitphasic disposition after intravenous injection. The initial distributive half-life of approximately 5 minutes suggests rapid tissue uptake of doxorubicin, while its slow elimination from tissues is reflected by a terminal half-life of 20 to 48 hours.

8.1.4 Supplier(s)

Doxorubicin is commercially available.

8.1.5 Dosage Form and Preparation

Doxorubicin for Injection is supplied as a sterile red-orange lyophilized powder in single dose flip-top vials in the following package strengths: 10 mg vial, 20 mg vial, 50 mg vial.

8.1.6 Storage and Stability

Store unreconstituted vial at 20º to 25º C. Retain in carton until time of use. Discard unused portion.

After adding the diluent, the vial should be shaken and the contents allowed to dissolve. The reconstituted solution is stable for 7 days at room temperature and under normal room light and 15 days under refrigeration (2º to 8º C). It should be protected from exposure to sunlight.

8.1.7 Administration

When possible, to reduce the risk of developing cardiotoxicity in patients receiving doxorubicin after stopping treatment with other cardiotoxic agents, especially those with long half-lives such as trastuzumab, doxorubicin-based therapy should be delayed until the other agents have cleared from the circulation.

Care in the administration of doxorubicin will reduce the chance of perivenous infiltration. It may also decrease the chance of local reactions such as urticaria and erythematous streaking. On intravenous administration of doxorubicin, extravasation may occur with or without an accompanying burning or stinging sensation, even if blood returns well on aspiration of the infusion needle. If any signs or symptoms of extravasation have occurred, the injection or infusion should be immediately terminated and restarted in another vein.

Doxorubicin will be administered during this study at a dose of 20 mg/m^2 weekly

for 6 weeks.

8.1.8 Special Handling Instructions

Caregivers should be counseled to take precautions (such as wearing latex gloves) to prevent contact with the patient's urine and other body fluids for at least 5 days after each treatment.

8.1.9 Expected Adverse Events

Doxorubicin may cause serious side effects including heart problems, secondary cancers (AML or MDS), and decreased blood cell counts (neutropenia, anemia, thrombocytopenia). It may also cause infusion site reactions (pain at injection site, skin redness or swelling, burning or stinging, open skin sores at injection site), change in the color of the patient's urine (red), infection, lower sperm counts and sperm problems, and irreversible amenorrhea or early menopause. The most common side effects of doxorubicin include: hair loss, darkening of the nails or separation of the nails from the nail bed, nausea, vomiting, lack of appetite or increased thirst, bruising or bleeding, abnormal heartbeat, secondary cancer, mouth sores, weight changes, stomach pain, diarrhea, eye problems, allergic reactions (rash, flushed face, fever, hives, dizziness, light-headedness, itching, shortness of breath, trouble breathing, swelling of the lips or tongue).

9.0 CORRELATIVE STUDIES

9.1 DSC-MRI

All patients in Arms B and C will undergo DSC-MRI at the following time points:

- no more than 2 weeks prior to MLA
- within approximately 3 days after MLA
- then every 2 weeks (+/- 3 days) for an additional 6 weeks
- 10 weeks (+/- 7 days) after MLA **only if** the 6-week scan shows prolonged disruption of the blood brain barrier
- 14 weeks (+/- 7 days) after MLA and then every 8 weeks (+/- 7 days) thereafter until disease progression

9.2 Blood for Serum Biomarkers of BBB Disruption

Patients will have 5 mL blood for serum and 2 mL blood for plasma drawn at the following time points:

- before biopsy/MLA (Arms A, B, and C); this sample can be collected any time during the 3 days before or the day of the procedure until the start of the procedure
- within approximately 3 days after biopsy/MLA (Arms A, B, and C)
- 1-2 weeks after biopsy (Arm A only)

- weekly (+/- 3 days) for 6 weeks (Arm B only)
- weekly (+/- 3 days) prior to (on the same day as) chemotherapy (Arm C only)
- weekly (+/- 3 days) within 24 hours after chemotherapy (Arm C only)
- 10 weeks (+/- 7 days) after MLA **only if** the 6-week post-MLA scan shows prolonged disruption of the blood brain barrier (Arms B and C)
- 14 weeks (+/- 7 days) after MLA and then every 8 weeks (+/- 7 days) until disease progression

For protein-based serum biomarker assays, 5 mL blood will be drawn into serum separator tubes (red cap or SST). SST tubes must be at least 50-80% full and should be mixed thoroughly. Immediately place samples on ice for 30-60 minutes to allow blood to clot. Samples will be transported to Tran lab in MDS 558, where samples will be centrifuged at 4000rpm for at 4°C for 30 minutes or until serum is completely separated. Serum is decanted, aliquoted and stored at -80°C until used for ELISA analysis.

For plasma microRNA-based assays, 2 mL blood will be drawn into an EDTA-containing tube (lavender cap). Tubes are inverted several times to mix and immediately placed on ice. Immediately transport samples to Tran lab, where samples will be centrifuged for 15 minutes at 2000g at 4°C. Plasma samples are removed, aliquoted, snap-frozen in liquid nitrogen, and stored at -80°C until used for microRNA analysis.

9.3 Blood for Phenotyping of Peripheral Blood Immune Cells

Patients in Arm A and the second 10 patients randomized to Arm B (but not the first 10 patients enrolled in Arm B) will have 40 mL of blood drawn before biopsy or biopsy + MLA and 20ml of blood drawn at indicated time points after biopsy or biopsy + MLA into green top tubes (with heparin) for comparison of immune profiles of peripheral blood cells before and after biopsy + MLA or biopsy alone.

Patients in Arm A will have blood drawn at the following time points:
- Approximately within 3 days before biopsy
- 2 weeks (+/- 3 days) and 4 weeks (+/- 3 days) after biopsy
- Every 2 weeks thereafter for up to 3 months after biopsy (provided there is no evidence of significant treatment-induced cytopenias)

Patients in Arm B will have blood drawn at the following time points:
- Approximately within 3 days before MLA
- 2 weeks (+/- 3 days) and 4 weeks (+/- 3 days) after biopsy
- Every 2 weeks thereafter for up to 3 months after biopsy (provided there is no evidence of significant treatment-induced cytopenias, defined as leukopenia and/or neutropenia that required growth factor support or persistent lymphopenia (ALC < 500 or CD4 < 200 for > 2 months) that requires chemoprophylaxis for opportunistic infections)

Blood will be Ficoll separated and the mononuclear cell population (PBMC) isolated per standard protocol[53,54]. For the PBMC isolated from 40ml of blood obtained prior to the

biopsy or biopsy + MLA, half will be cryopreserved for later analysis; the other half will be used to isolate monocytes per standard protocol[54]. The isolated monocytes will be incubated with IL-4 and GM-CSF to produce dendritic cells (DC)[54] which will then be cryopreserved for analysis of tumor-specific functional activity of peripheral blood immune cells detailed in section 9.4 below. To determine the phenotype of peripheral blood immune cells, following the 4-week interval, cryopreserved PBMC will be thawed and stained with labeled antibodies to CD4, CD8, CD45, CCR7, CD27, CD28, and CD62, as well as to markers of T cell hypofunctionality (PD-1, CTLA-4, LAG-3, TIM3, and ICOS). Specifically, we will compare levels of $CD8^+PD\text{-}1^+LAG\text{-}3^+$ T cells between MLA-treated and untreated patients, as this cell population has been shown to mark tumor-specific $CD8^+$ T cells. Cells will be assessed by flow cytometry in the CHiiPs immune monitoring core.

9.4 Biopsy for Tumor-Specific Functional Activity of Peripheral Blood Immune Cells

The 5 patients in Arm A and the second 10 patients randomized to Arm B (but not the first 10 patients enrolled in Arm B) will have a biopsy either alone or immediately before MLA, respectively. Specimens not used for histologic diagnosis will be cryopreserved. After PBMC samples of 10 Arm B and 5 Arm A patients have been obtained, we will test T cell reactivity in an in vitro stimulation assay. Dendritic cells matured from monocytes as described in Section 9.3 above will be incubated with freeze-thawed biopsy specimen lysate for 2 days and subsequently incubated with T cells purified from PBMC using CD3/CD8-positive microbeads. Five days after stimulation, T cell reactivity will be assessed by ELISPOT assay for IFN-γ stimulation. This study will test whether MLA stimulates increased tumor-specific T cell recognition and activation.

9.5 Quality of Life Assessments

Patients in Arms B and C will undergo QOL assessments in the form of evaluation of Karnofsky performance status and MMSE (Appendix 2) at the following time points:

- baseline
- Week 6 for Arm B patients and Week 7 for Arm C patients
- Week 14
- every 8 weeks thereafter, following the DSC-MRI schedule, until disease progression

10.0 STUDY CALENDARS

10.1 Arm A Study Calendar

Baseline evaluations are to be conducted within 2 weeks prior to start of protocol procedures unless otherwise noted.

	Baseline	Biopsy	3 Days Post-Bx	2 Wks Post-Bx[1]	4 Wks Post-Bx[1]	Every 2 Wks Thereafter for 3 Mos Post-Bx	F/U[4]
Informed consent	X						
Biopsy		X					
Blood to measure brain-specific factors (Section 9.2)		X[2]	X	X	X		
Blood for dendritic cells and PBMC (Section 9.3)		X[2]		X	X	X[3]	
Adverse event assessment		X[5]	X[5]	X[5]	X[5]	X[5]	X[5]

1. +/3 days
2. May be collected any time during the 3 days before or the day of the procedure until the start of the procedure
3. If the leukocyte counts are inadequate, the blood draw will be skipped at that particular time point. If the leukocyte counts remain inadequate for more than 4 weeks, the blood draw will be discontinued and the patient will cease to participate in the study.
4. To take place 7-14 days after the final blood samples are obtained to determine if the patient experienced any reportable adverse events.
5. Evaluating for adverse events considered possibly, probably, or definitely related to the blood draws only.

10.2 Arm B Study Calendar

Baseline evaluations are to be conducted within 2 weeks prior to the start of protocol therapy unless otherwise noted.

	B/L	2 Wks Pre-MLA	MLA	3 Days Post-MLA	Wk 1[8]	Wk 2[8]	Wk 3[8]	Wk 4[8]	Wk 5[8]	Wk 6[8]	Wk 7[8]	Wk 8[8]	Wk 9[8]	Wk 10	Wk 11[8]	Wk 12[8]	Wk 14[10]	F/U[16]
Informed consent	X																	
Medical history	X																	
Physical exam incl. wt	X									X		X		X[8]			X	X[11,14]
Karnofsky PS, MMSE	X									X							X	X[11,14]
CBC[1]	X									X	X	X	X	X[8]	X	X		
CMP[1]	X									X			X			X		
LFTs[2]											X	X		X[8]	X			
β-hCG[3]	X																	
Echo or MUGA[4]	X																	
DSC-MRI		X[5]		X		X		X		X				X[9,10]			X	X[10,11]
Blood to measure brain-specific factors (Section 9.2)			X[6]	X	X	X	X	X	X	X				X[9,10]			X	X[10,11]
Blood for dendritic cells and PBMC (Section 9.3)[7]			X[6]			X		X		X[12]		X[12]		X[8,12]		X[12]		
Doxorubicin[13]											X	X	X	X[8]	X	X		
AE assessment[15]	X -- X																	

1. No more than 7 days prior to MLA.
2. AST, ALT, bilirubin, alkaline phosphatase.
3. Women of childbearing potential only.
4. No more than 1 year prior to registration. Only for patients with histories of CHF and/or CAD requiring Rx other than aspirin, or a history of exposure to anthracyclines.
5. May take place any time between MLA and 14 days prior to MLA.
6. May be collected any time during the 3 days before or the day of the procedure until the start of the procedure.
7. Only drawn from the second group of 10 patients enrolled to Arm B (those randomized to Arm B).
8. +/- 3 days
9. Only if the 6-week scan showed prolonged BBB disruption
10. +/- 7 days
11. Every 8 weeks until disease progression.
12. If the leukocyte counts are inadequate, the blood draw will be skipped at that particular time point. If the leukocyte counts remain inadequate for more than 4 weeks, the blood draw will be discontinued.
13. May be initiated 6-8 weeks after MLA; this calendar shows initiation at 6 weeks after MLA.
14. To coincide with DSC-MRI.
15. Through 30 days after last dose of doxorubicin
16. Patients will be followed every 8 weeks from the Week 6 DSC-MRI until 2 years have elapsed since patient registration or death, whichever occurs first. After progression, vital status and subsequent treatment for GBM will be recorded.

10.3 Arm C Study Calendar

Baseline evaluations are to be conducted within 2 weeks prior to the start of protocol therapy unless otherwise noted.

	B/L	2 Wks Pre-MLA	MLA	3 Days Post-MLA	Wk 1[8]	Wk 2[8]	Wk 3[8]	Wk 4[8]	Wk 5[8]	Wk 6[8]	Wk 7[8]	Wk 8	Wk 9	Wk 10[10]	Wk 11	Wk 12	Wk 14[10]	F/U[16]
Informed consent	X																	
Medical history	X																	
Physical exam incl. wt	X				X		X		X		X						X	X[11,14]
Karnofsky PS, MMSE	X										X						X	X[11,14]
CBC[1]	X				X	X	X	X	X	X	X							
CMP[1]	X				X			X			X							
LFTs[2]						X	X		X	X								
β-hCG[3]	X																	
Echo or MUGA[4]	X																	
DSC-MRI		X[5]		X		X		X		X				X[9]			X	X[10,11]
Blood to measure brain-specific factors (Section 9.2)			X[6]	X	X[12]	X[12]	X[12]	X[12]	X[12]	X[12]				X[9]			X	X[10,11]
Doxorubicin[13]					X	X	X	X	X	X								
AE assessment[15]	X -- X																	

1. No more than 7 days prior to MLA.
2. AST, ALT, bilirubin, alkaline phosphatase.
3. Women of childbearing potential only.
4. No more than 1 year prior to registration. Only for patients with histories of CHF and/or CAD requiring Rx other than aspirin, or a history of exposure to anthracyclines.
5. May take place any time between MLA and 14 days prior to MLA.
6. May be collected any time during the 3 days before or the day of the procedure until the start of the procedure.
7. Only drawn from the second group of 10 patients enrolled to Arm B (those randomized to Arm B).
8. +/- 3 days
9. Only if the 6-week scan showed prolonged BBB disruption
10. +/- 7 days
11. Every 8 weeks until disease progression.
12. Same day as chemotherapy and again 24 hours after chemotherapy.
13. To begin approximately 7 days after MLA.
14. To coincide with DSC-MRI.
15. Through 30 days after last dose of doxorubicin
16. Patients will be followed every 8 weeks from the Week 6 DSC-MRI until 2 years have elapsed since patient registration or death, whichever occurs first. After progression, vital status and subsequent treatment for GBM will be recorded.

11.0 DATA SUBMISSION SCHEDULE

Case report forms with appropriate source documentation will be completed within 60 days of the schedule listed in this section.

Case Report Form	Submission Schedule
Original Consent Form	Prior to registration
Registration Form<br>Eligibility Form<br>On-Study Form	Prior to starting treatment
Biopsy Form (Arm A only)<br>MLA Form (Arms B and C only)	After procedure
DSC-MRI Form	Weeks 2, 4, and 6<br>Week 10 (if applicable) and 14
Biomarkers Form	Pre- and post-MLA<br>Weeks 1 through 6<br>Week 10 (if applicable) and 14
Toxicity Form	Continuous through 30 days after the end of doxorubicin (Arms B and C)<br>Through 7 days after last blood draw (Arm A)
Treatment Summary Form	Completion of treatment
Follow Up Form (Arms B and C only)	Every month for the first year and every two months for the second year
QOL Form (Arms B and C only)	Baseline<br>Week 6<br>Week 12<br>Every 8 weeks thereafter through the end of follow-up
Tumor Measurement Form	Pre- and post-MLA<br>Week 6<br>Week 14<br>Every 8 weeks thereafter through the end of follow-up
Adverse Events Form	See Section 7.0 for reporting requirements

12.0 MEASUREMENT OF EFFECT

12.1 Antitumor Effect – Solid Tumors

For the purposes of this study, patients should be re-evaluated for response every 8 weeks. The DSC-MRI scan obtained within approximately 3 days after MLA will be used as the baseline scan for determining response. The DSC-MRI scans obtained at pre-MLA, 2 weeks post-MLA, 4 weeks post-MLA, 6 weeks post-MLA, and 10 weeks post-MLA (if obtained) will not be assessed for disease response. In addition to a baseline scan, confirmatory scans should also be obtained 8 weeks (not less than 4) weeks following initial documentation of objective response.

Response and progression will be evaluated in this study using the updated response assessment criteria for high-grade gliomas: Response Assessment in Neuro-Oncology (RANO) working group guideline [JCO 28(11): 1963-1972, 2010].

Criteria for Determining First Progression Depending on Time From Initial Chemoradiotherapy

First Progression	Definition
Progressive disease < 12 weeks after completion of chemoradiotherapy	Progression can only be defined using diagnostic imaging if there is new enhancement outside of the radiation field (beyond the high-dose region or 80% isodose line) or if there is unequivocal evidence of viable tumor on histopathologic sampling (eg, solid tumor areas [ie, > 70% tumor cell nuclei in areas], high or progressive increase in MIB-1 proliferation index compared with prior biopsy, or evidence for histologic progression or increased anaplasia in tumor). Note: Given the difficulty of differentiating true progression from pseudoprogression, clinical decline alone, in the absence of radiographic or histologic confirmation of progression, will not be sufficient for definition of progressive disease in the first 12 weeks after completion of concurrent chemoradiotherapy.
Progressive disease ≥ 12 weeks after chemoradiotherapy completion	1. New contrast-enhancing lesion outside of radiation field on decreasing, stable, or increasing doses of corticosteroids.<br>2. Increase by ≥ 25% in the sum of the products of perpendicular diameters between the first postradiotherapy scan, or a subsequent scan with smaller tumor size, and the scan at 12 weeks or later on stable or increasing doses of corticosteroids.<br>3. Clinical deterioration not attributable to concurrent medication or comorbid conditions is sufficient to declare progression on current treatment but not for entry onto a clinical trial for recurrence.<br>4. For patients receiving antiangiogenic therapy, significant increase in T2/FLAIR nonenhancing lesion may also be considered progressive disease. The increased T2/FLAIR must have occurred with the patient on stable or increasing doses of corticosteroids compared with baseline scan or best response after initiation of therapy and not be a result of comorbid events (eg, effects of radiation therapy, demyelination, ischemic injury, infection, seizures, postoperative changes, or other treatment effects).

Criteria for Response Assessment Incorporating MRI and Clinical Factors (Adapted from JCO 2010)

Response	Criteria
Complete response	• Requires all of the following: complete disappearance of all enhancing measurable and nonmeasurable disease sustained for at least 4 weeks.<br>• No new lesions; stable or improved nonenhancing (T2/FLAIR) lesions.<br>• Patients must be off corticosteroids (or on physiologic replacement doses only) and stable or improved clinically. Note: Patients with nonmeasurable disease only cannot have a complete response; the best response possible is stable disease.
Partial response	Requires all of the following:<br>• ≥ 50% decrease compared with baseline in the sum of products of perpendicular diameters of all measurable enhancing lesions sustained for at least 4 weeks.<br>• No progression of nonmeasurable disease.<br>• Any new measureable lesion within the 3 cm radius of the rim of the MLA-treated recurrent tumor. †<br>• Stable or improved nonenhancing (T2/FLAIR) lesions on same or lower dose of corticosteroids compared with baseline scan; the corticosteroid dose at the time of the scan evaluation should be no greater than the dose at time of baseline scan.<br>• Stable or improved clinically. Note: Patients with nonmeasurable disease only cannot have a partial response; the best response possible is stable disease.
Stable disease	Requires all of the following:<br>• Does not qualify for complete response, partial response, or progression.<br>• Stable nonenhancing (T2/FLAIR) lesions on same or lower dose of corticosteroids compared with baseline scan. In the event that the corticosteroid dose was increased for new symptoms and signs without confirmation of disease progression on neuroimaging, and subsequent follow-up imaging shows that this increase in corticosteroids was required because of disease progression, the last scan considered to show stable disease will be the scan obtained when the corticosteroid dose was equivalent to the baseline dose.
Progression	Defined by any of the following:<br>• ≥ 25% increase in sum of the products of perpendicular diameters of enhancing lesions compared with the smallest tumor measurement obtained either at baseline (if no decrease) or best response, on stable or increasing doses of corticosteroids*.<br>• Significant increase in T2/FLAIR nonenhancing lesion on stable or increasing doses of corticosteroids compared with baseline scan or best response after initiation of therapy* not caused by comorbid events (e.g. radiation therapy, demyelination, ischemic injury, infection, seizures,

Response	Criteria
	postoperative changes, or other treatment effects).<br>• Any new measureable lesion within the 3 cm radius of the rim of the MLA-treated recurrent tumor. †<br>• Clear clinical deterioration not attributable to other causes apart from the tumor (e.g. seizures, medication adverse effects, complications of therapy, cerebrovascular events, infection, and so on) or changes in corticosteroid dose.<br>• Failure to return for evaluation as a result of death or deteriorating condition; or clear progression of nonmeasurable disease.

- NOTE. All measurable and nonmeasurable lesions must be assessed using the same techniques as at baseline.
- Abbreviations: MRI, magnetic resonance imaging; FLAIR, fluid-attenuated inversion recovery.
- * Stable doses of corticosteroids include patients not on corticosteroids.
- † If any new measurable lesion is outside the 3 cm radius of the rim of the MLA-treated recurrent tumor or is located in the contralateral hemisphere regardless of its distance from the rim of the MLA-treated tumor AND there is not any measurable lesion within the 3 cm radius, the patient will be considered to have progressive disease but will not be considered evaluable for the purpose of this study, in which only local disease control or failure is measured.

12.2 Disease Parameters

Measurable disease: Bi-dimensionally measurable lesions with clearly defined margins by MRI scan. All tumor measurements must be recorded in millimeters (or decimal fractions of centimeters).

Non-measurable or evaluable disease: Uni-dimensionally measurable lesions or lesions with margins not clearly defined such as areas of T2/FLAIR signal abnormality or poorly defined enhancing abnormality.

Note: For cystic lesions, the only measurable part is any enhancement area around the cyst that is clearly defined and bi-dimensionally measurable. The cyst itself should not be considered measurable or non-measureable disease.

Target lesions: All measurable lesions that are residual of the lesion treated with MLA or that are located within the 3 cm radius of the rim of the MLA-treated recurrent tumor should be identified as target lesions and recorded and measured. Target lesions should be selected on the basis of their size (lesions with the longest diameter), but in addition should be those that lend themselves to reproducible repeated measurements. It may be the case that, on occasion, the largest lesion does not lend itself to reproducible measurement in which circumstance the next largest lesion, which can be measured reproducibly should be selected. When there are too many measurable lesions, choose

the largest 3 lesions as target lesions to follow. The other measurable lesions should be considered evaluable for the purpose of objective status determination.

Non-target lesions: All non-measurable lesions should be identified as non-target lesions and should also be recorded at baseline. Measurements of these lesions are not required, but the presence, absence, or in rare cases unequivocal progression of each should be noted throughout follow-up.

12.3 Methods for Evaluation of Measurable Disease

All measurements should be taken and recorded in metric notation using a ruler. All baseline evaluations should be performed as closely as possible to the beginning of treatment and never more than 2 weeks before the beginning of the treatment.

Clinical lesions: Clinical lesions will only be considered measurable on brain MRI when they are ≥ 5 mm diameter as assessed using a ruler.

Histology: This technique can be used to differentiate between partial responses (PR) and complete responses (CR) in rare cases when biopsy or surgical resection of a measureable lesion is clinically indicated.

Perfusion/CBV: This advanced brain MRI technique can be used as an adjunct test to determine treatment response or disease status. However, it should not be used as the primary or sole method to determine response or disease status.

Brain FDG-PET coupled with head CT or brain MRI: This advanced metabolic imaging technique can be used as an adjunct test to determine response or disease status. However it should be used as the primary or sole method of determining response or disease status.

12.3.1 Evaluation of Target Lesions

Complete Response (CR): Disappearance of all target lesions.

Partial Response (PR): ≥ 50% decrease compared with baseline in the sum of products of perpendicular diameters of all target lesions sustained for at least 4 weeks.

Progressive Disease (PD): At least a 25% increase in the sum of products of perpendicular diameters of at least 1 target lesion, taking as reference the smallest sum of products of perpendicular diameters on study (this includes the baseline sum if that is the smallest on study).

Stable Disease (SD): Neither sufficient shrinkage to qualify for PR nor sufficient increase to qualify for PD, taking as reference the smallest sum of products of perpendicular diameters while on study.

12.3.2 Evaluation of Non-Target Lesions

Complete Response (CR): Disappearance of all non-target lesions.

Non-CR/Non-PD: Persistence of one or more non-target lesion(s).

Progressive Disease (PD): Appearance of one or more new lesions and/or unequivocal progression of existing non-target lesions on stable or increasing doses of corticosteroids compared with baseline scan or best response after initiation of therapy* not caused by comorbid events (e.g. radiation therapy, demyelination, ischemic injury, infection, seizures, postoperative changes, or other treatment effects). Unequivocal progression should not normally trump target lesion status. It must be representative of overall disease status change, not a single lesion increase.

Although a clear progression of "non-target" lesions only is exceptional, the opinion of the treating physician should prevail in such circumstances, and the progression status should be confirmed at a later time by the review panel (or Principal Investigator).

12.3.3 Evaluation of Best Overall Response

The best overall response is the best response recorded from the start of the treatment until disease progression/recurrence (taking as reference for progressive disease the smallest measurements recorded since the treatment started). The patient's best response assignment will depend on the achievement of both measurement and confirmation criteria.

Summary of the RANO Response Criteria (Adapted from JCO 2010)

Criterion	CR	PR	SD	PD
T1 gadolinium enhancing disease	None	≥ 50% ↓	< 50% ↓ but < 25% ↑	≥ 25% ↑*
T2/FLAIR	Stable or ↓	Stable or ↓	Stable or ↓	↑*
New lesion	None	None	None	Present*
Corticosteroids	None	Stable or ↓	Stable or ↓	NA†
Clinical status	Stable or ↑	Stable or ↑	Stable or ↑	↓*
Requirement for response	All	All	All	Any*

Abbreviations: RANO, Response Assessment in Neuro-Oncology; CR, complete response; PR, partial response; SD, stable disease; PD, progressive disease; FLAIR, fluid-attenuated inversion recovery; NA, not applicable.
* Progression occurs when this criterion is present.
† Increase in corticosteroids alone will not be taken into account in determining progression in the absence of persistent clinical deterioration.

12.3.4 Duration of Response

Duration of overall response: The duration of overall response is measured from the time measurement criteria are met for CR or PR (whichever is first recorded) until the first date that recurrent or progressive disease is objectively documented (taking as reference for progressive disease the smallest measurements recorded since the treatment started).

The duration of overall CR is measured from the time measurement criteria are first met for CR until the first date that progressive disease is objectively documented.

Duration of stable disease: Stable disease is measured from the start of the treatment until the criteria for progression are met, taking as reference the smallest measurements recorded since the treatment started, including the baseline measurements.

12.3.5 Neurological Exam and Performance Status

Patients will be graded using the Karnofsky Performance Status scale and their neurological function evaluated as improved, stable or deteriorated in addition to objective measurement of tumor size. These parameters will be used to determine the overall response assessment.

12.3.6 Progression-Free Survival

PFS is defined as the duration of time from start of treatment to time of progression or death, whichever occurs first.

13.0 DATA AND SAFETY MONITORING

In compliance with the Washington University Institutional Data and Safety Monitoring Plan, the Principal Investigator will provide a Data and Safety Monitoring (DSM) report to the Washington University Quality Assurance and Safety Monitoring Committee (QASMC) semi-annually beginning six months after accrual has opened (if at least five patients have been enrolled) or one year after accrual has opened (if fewer than five patients have been enrolled at the six-month mark).

The Principal Investigator will review all patient data at least every six months, and provide a semi-annual report to the QASMC. This report will include:

- HRPO protocol number, protocol title, Principal Investigator name, data coordinator name, regulatory coordinator name, and statistician
- Date of initial HRPO approval, date of most recent consent HRPO approval/revision, date of HRPO expiration, date of most recent QA audit, study status, and phase of study

- History of study including summary of substantive amendments; summary of accrual suspensions including start/stop dates and reason; and summary of protocol exceptions, error, or breach of confidentiality including start/stop dates and reason
- Study-wide target accrual and study-wide actual accrual
- Protocol activation date
- Average rate of accrual observed in year 1, year 2, and subsequent years
- Expected accrual end date and accrual by arm
- Objectives of protocol with supporting data and list the number of participants who have met each objective
- Measures of efficacy
- Early stopping rules with supporting data and list the number of participants who have met the early stopping rules
- Summary of toxicities separated by arm
- Abstract submissions/publications
- Summary of any recent literature that may affect the safety or ethics of the study

The study principal investigator and Research Patient Coordinator will monitor for serious toxicities on an ongoing basis. Once the principal investigator or Research Patient Coordinator becomes aware of an adverse event, the AE will be reported to the HRPO and QASMC according to institutional guidelines.

14.0 STATISTICAL CONSIDERATIONS

Since we do not know which biomarkers will have better correlation with the Ktrans data from DSC-MRI and patients' survival outcome, we plan to determine the levels of all 4 biomarkers in a blinded fashion. Once both the Ktrans and biomarker levels are available, we will determine which biomarkers have the closest correlation that is statistically significant with the Ktrans. Pearson correlation coefficient (r) will be determined for each biomarker and Ktrans value. Biomarkers with higher correlation coefficient (r approaching 1) will be given higher priority. A minimum r=0.5 is required for inclusion for further analysis and will be used as a peritumoral permeability score. This score will then be correlated with the patient outcome data (as measured by 6-PFS rate) to determine whether it has a predictive value.

Since this is a pilot study, it is not feasible to estimate sample size for overall survival. Therefore, our sample size calculation is based on PFS. Single-agent bevacizumab has a reported 6-PFS rate of 42.6%[4]. Our study will have 24-month accrual and 2-year follow-up. Using a log-rank test with one-side 0.05 and 80% power, we will need a sample size of 29 evaluable per group if the 6-PFS in the experimental arm is 65%. If the experimental arm's 6-PFS is 70%, we will need 19 evaluable patients per group. Based on our previous experiences with MLA followed by early chemotherapy suggesting that PFS is likely greater than 6 months (Table 1), we think these accrual projections are within reason. We will conduct an interim analysis after 10 evaluable subjects are enrolled in each arm before proceeding with the planned accrual of 20 evaluable per arm. Overall survival will be analyzed using a log-rank test.

Table 1<br>MLA Experience in Patients with GBM at<br>Siteman Cancer Center in the Past 12 Months					
Age at MLA	Diagnosis	Date of MLA	Chemotherapy (<3 wks after MLA)	PFS (months)	Alive
1 73	GBM	11-4-11	Yes	12	Yes
2 64	GBM	12-27-11	Yes	10	Yes
3 34	High grade, likely GBM	01-03-12	Yes	Not yet progressed	Yes
4 46	High grade, likely GBM	01-16-12	Yes	11	Yes
5 72	GBM	02-10-12	Yes	Not yet progressed	Yes
6 68	GBM	07-06-12	Yes	Not yet progressed	Yes

If our hypothesis is correct, we expect to see a much greater 6-PFS rate in the early chemotherapy arm as compared to the late chemotherapy arm and the historical control (bevacizumab alone). Since the study is powered to detect a difference between one of these two arms and the historical control, a larger sample size may be required to detect a difference between these two arms themselves. If the window of BBB leakage extends beyond 6 weeks post MLA, we will delay chemotherapy in the control arm to a maximum of 8 weeks after MLA out of concern for patients' safety, recognizing that this may confound the results. In the control arm, the delay of doxorubicin for 6 weeks may bias against this arm. Although possible, this bias is unlikely since it has been shown that delaying chemotherapy for up to 6 weeks after surgery did not negatively impact survival outcomes[55]. The weekly dosing of low dose doxorubicin was chosen based on its pharmacokinetics and better tolerability. However, if the difference between the two arms and the historical control is not detected, we will use other dosing schedules (e.g. 40-60 mg/m^2 every 3 weeks) to achieve adequate drug concentration even with BBB disruption.

15.0 REFERENCE

1. Parkin, D.M. Global cancer statistics in the year 2000. *The Lancet Oncology* **2**, 533-543 (2001).
2. Stupp, R., *et al.* Radiotherapy plus Concomitant and Adjuvant Temozolomide for Glioblastoma. *New England Journal of Medicine* **352**, 987-996 (2005).
3. Stupp, R., *et al.* Effects of radiotherapy with concomitant and adjuvant temozolomide versus radiotherapy alone on survival in glioblastoma in a randomised phase III study: 5-year analysis of the EORTC-NCIC trial. *The Lancet Oncology* **10**, 459-466 (2009).
4. Lai, A., *et al.* Phase II Study of Bevacizumab Plus Temozolomide During and After Radiation Therapy for Patients With Newly Diagnosed Glioblastoma Multiforme. *Journal of Clinical Oncology* **29**, 142-148 (2011).
5. Ananthnarayan, S., *et al.* Time course of imaging changes of GBM during extended bevacizumab treatment. *Journal of neuro-oncology* **88**, 339-347 (2008).
6. Bergers, G. & Hanahan, D. Modes of resistance to anti-angiogenic therapy. *Nat Rev Cancer* **8**, 592-603 (2008).
7. Adamson, C., *et al.* Glioblastoma multiforme: a review of where we have been and where we are going. *Expert opinion on investigational drugs* **18**, 1061-1083 (2009).
8. Holodny, A.I., *et al.* Correlation between the degree of contrast enhancement and the volume of peritumoral edema in meningiomas and malignant gliomas. *Neuroradiology* **41**, 820-825 (1999).
9. Kassner, A. & Thornhill, R. Measuring the integrity of the human blood-brain barrier using magnetic resonance imaging. *Methods in molecular biology* **686**, 229-245 (2011).
10. Rosner, D., Nemoto, T. & Lane, W.W. Chemotherapy induces regression of brain metastases in breast carcinoma. *Cancer* **58**, 832-839 (1986).
11. Lee, J.S., *et al.* Primary chemotherapy of brain metastasis in small-cell lung cancer. *J Clin Oncol* **7**, 916-922 (1989).
12. Postmus, P.E., *et al.* Treatment of Brain Metastases of Small-Cell Lung Cancer: Comparing Teniposide and Teniposide With Whole-Brain Radiotherapy,ÄîA Phase III Study of the European Organization for the Research and Treatment of Cancer Lung Cancer Cooperative Group. *Journal of Clinical Oncology* **18**, 3400-3408 (2000).
13. Fujita, A., Fukuoka, S., Takabatake, H., Tagaki, S. & Sekine, K. Combination Chemotherapy of Cisplatin, Ifosfamide, and Irinotecan with rhG-CSF Support in Patients with Brain Metastases from Non-Small Cell Lung Cancer. *Oncology* **59**, 291-295 (2000).
14. Bernardo, G., *et al.* First-Line Chemotherapy with Vinorelbine, Gemcitabine, and Carboplatin in the Treatment of Brain Metastases from Non-Small-Cell Lung Cancer: A Phase II Study. *Cancer Investigation* **20**, 293-302 (2002).
15. Korfel, A., *et al.* Response to topotecan of symptomatic brain metastases of small-cell lung cancer also after whole-brain irradiation: a multicentre phase II study. *European Journal of Cancer* **38**, 1724-1729 (2002).
16. Deeken, J.F. & L√∂scher, W. The Blood-Brain Barrier and Cancer: Transporters, Treatment, and Trojan Horses. *Clinical Cancer Research* **13**, 1663-1674 (2007).
17. Stewart, D.J., *et al.* Penetration of VP-16 (etoposide) into human intracerebral and extracerebral tumors. *Journal of neuro-oncology* **2**, 133-139 (1984).
18. Stewart, D.J., *et al.* Concentration of vinblastine in human intracerebral tumor and other tissues. *Journal of neuro-oncology* **1**, 139-144 (1983).

19. Hochberg, F.H. & Pruitt, A. Assumptions in the radiotherapy of glioblastoma. *Neurology* **30**, 907-911 (1980).
20. Brem, H., *et al.* Placebo-controlled trial of safety and efficacy of intraoperative controlled delivery by biodegradable polymers of chemotherapy for recurrent gliomas. The Polymer-brain Tumor Treatment Group. *Lancet* **345**, 1008-1012 (1995).
21. Westphal, M., Ram, Z., Riddle, V., Hilt, D. & Bortey, E. Gliadel wafer in initial surgery for malignant glioma: long-term follow-up of a multicenter controlled trial. *Acta neurochirurgica* **148**, 269-275; discussion 275 (2006).
22. Westphal, M., *et al.* A phase 3 trial of local chemotherapy with biodegradable carmustine (BCNU) wafers (Gliadel wafers) in patients with primary malignant glioma. *Neuro-oncology* **5**, 79-88 (2003).
23. Fleming, A.B. & Saltzman, W.M. Pharmacokinetics of the carmustine implant. *Clinical pharmacokinetics* **41**, 403-419 (2002).
24. Bobo, R.H., *et al.* Convection-enhanced delivery of macromolecules in the brain. *Proceedings of the National Academy of Sciences* **91**, 2076-2080 (1994).
25. Hawasli, A.H., Ray, W.Z., Murphy, R.K., Dacey, R.G., Jr. & Leuthardt, E.C. Magnetic resonance imaging-guided focused laser interstitial thermal therapy for subinsular metastatic adenocarcinoma: technical case report. *Neurosurgery* **70**, 332-337; discussion 338 (2012).
26. Gong, W., *et al.* Improving efficiency of adriamycin crossing blood brain barrier by combination of thermosensitive liposomes and hyperthermia. *Biological & pharmaceutical bulletin* **34**, 1058-1064 (2011).
27. Oztas, B. & Kucuk, M. Reversible blood-brain barrier dysfunction after intracarotid hyperthermic saline infusion. *International journal of hyperthermia : the official journal of European Society for Hyperthermic Oncology, North American Hyperthermia Group* **14**, 395-401 (1998).
28. Sabel, M., *et al.* Locoregional opening of the rodent blood-brain barrier for paclitaxel using Nd:YAG laser-induced thermo therapy: a new concept of adjuvant glioma therapy? *Lasers in surgery and medicine* **33**, 75-80 (2003).
29. Wolff, J.E.A., Trilling, T., Mölenkamp, G., Egeler, R.M. & Jürgens, H. Chemosensitivity of glioma cells in vitro: a meta analysis. *Journal of Cancer Research and Clinical Oncology* **125**, 481-486 (1999).
30. Ostergaard, L., *et al.* High resolution measurement of cerebral blood flow using intravascular tracer bolus passages. Part II: Experimental comparison and preliminary results. *Magn Reson Med* **36**, 726-736 (1996).
31. Donahue, K.M., *et al.* Utility of simultaneously acquired gradient-echo and spin-echo cerebral blood volume and morphology maps in brain tumor patients. *Magn Reson Med* **43**, 845-853 (2000).
32. Johnson, G., Wetzel, S.G., Cha, S., Babb, J. & Tofts, P.S. Measuring blood volume and vascular transfer constant from dynamic, T(2)*-weighted contrast-enhanced MRI. *Magn Reson Med* **51**, 961-968 (2004).
33. Law, M., *et al.* Comparing perfusion metrics obtained from a single compartment versus pharmacokinetic modeling methods using dynamic susceptibility contrast-enhanced perfusion MR imaging with glioma grade. *AJNR Am J Neuroradiol* **27**, 1975-1982 (2006).

34. Blyth, B.J., *et al.* Validation of serum markers for blood-brain barrier disruption in traumatic brain injury. *Journal of neurotrauma* **26**, 1497-1507 (2009).
35. Correale, J., *et al.* Status epilepticus increases CSF levels of neuron-specific enolase and alters the blood-brain barrier. *Neurology* **50**, 1388-1391 (1998).
36. Foerch, C., *et al.* Serum glial fibrillary acidic protein as a biomarker for intracerebral haemorrhage in patients with acute stroke. *Journal of neurology, neurosurgery, and psychiatry* **77**, 181-184 (2006).
37. Haufschild, T., Shaw, S.G., Kesselring, J. & Flammer, J. Increased endothelin-1 plasma levels in patients with multiple sclerosis. *Journal of neuro-ophthalmology : the official journal of the North American Neuro-Ophthalmology Society* **21**, 37-38 (2001).
38. Husain, H., *et al.* Pre- and post-operative plasma glial fibrillary acidic protein levels in patients with newly diagnosed gliomas. *Journal of neuro-oncology* **109**, 123-127 (2012).
39. Kanner, A.A., *et al.* Serum S100beta: a noninvasive marker of blood-brain barrier function and brain lesions. *Cancer* **97**, 2806-2813 (2003).
40. Kapural, M., *et al.* Serum S-100beta as a possible marker of blood-brain barrier disruption. *Brain research* **940**, 102-104 (2002).
41. Marchi, N., *et al.* Peripheral markers of brain damage and blood-brain barrier dysfunction. *Restorative neurology and neuroscience* **21**, 109-121 (2003).
42. Pham, N., *et al.* Extracranial sources of S100B do not affect serum levels. *PloS one* **5**(2010).
43. Schiff, L., Hadker, N., Weiser, S. & Rausch, C. A literature review of the feasibility of glial fibrillary acidic protein as a biomarker for stroke and traumatic brain injury. *Molecular diagnosis & therapy* **16**, 79-92 (2012).
44. Laterza, O.F., *et al.* Plasma MicroRNAs as sensitive and specific biomarkers of tissue injury. *Clinical chemistry* **55**, 1977-1983 (2009).
45. Majetschak, M., *et al.* Circulating proteasomes after burn injury. *Journal of burn care & research : official publication of the American Burn Association* **31**, 243-250 (2010).
46. Dunn, G.P., Dunn, I.F. & Curry, W.T. Focus on TILs: Prognostic significance of tumor infiltrating lymphocytes in human glioma. *Cancer Immun* **7**, 12 (2007).
47. Claes, A., *et al.* Antiangiogenic compounds interfere with chemotherapy of brain tumors due to vessel normalization. *Mol Cancer Ther* **7**, 71-78 (2008).
48. Thompson, E.M., Frenkel, E.P. & Neuwelt, E.A. The paradoxical effect of bevacizumab in the therapy of malignant gliomas. *Neurology* **76**, 87-93 (2011).
49. Dix, A.R., Brooks, W.H., Roszman, T.L. & Morford, L.A. Immune defects observed in patients with primary malignant brain tumors. *J Neuroimmunol* **100**, 216-232 (1999).
50. Dunn, G.P., Fecci, P.E. & Curry, W.T. Cancer immunoediting in malignant glioma. *Neurosurgery* **71**, 201-222; discussion 222-203 (2012).
51. Fecci, P.E., *et al.* Increased regulatory T-cell fraction amidst a diminished CD4 compartment explains cellular immune defects in patients with malignant glioma. *Cancer Res* **66**, 3294-3302 (2006).
52. Okada, H., *et al.* Induction of CD8+ T-cell responses against novel glioma-associated antigen peptides and clinical activity by vaccinations with {alpha}-type 1 polarized dendritic cells and polyinosinic-polycytidylic acid stabilized by lysine and carboxymethylcellulose in patients with recurrent malignant glioma. *J Clin Oncol* **29**, 330-336 (2011).

53. Flomenberg, P., Piaskowski, V., Truitt, R.L. & Casper, J.T. Characterization of human proliferative T cell responses to adenovirus. *J Infect Dis* **171**, 1090-1096 (1995).
54. Tang, J., Flomenberg, P., Harshyne, L., Kenyon, L. & Andrews, D.W. Glioblastoma patients exhibit circulating tumor-specific CD8+ T cells. *Clin Cancer Res* **11**, 5292-5299 (2005).
55. Lai, R., Hershman, D.L., Doan, T. & Neugut, A.I. The timing of cranial radiation in elderly patients with newly diagnosed glioblastoma multiforme. *Neuro-oncology* **12**, 190-198 (2010).

APPENDIX 1: Karnofsky Performance Scale

Able to carry on normal activity and to work; no special care needed.	100	Normal no complaints; no evidence of disease.
	90	Able to carry on normal activity; minor signs or symptoms of disease.
	80	Normal activity with effort; some signs or symptoms of disease.
Unable to work; able to live at home and care for most personal needs; varying amount of assistance needed.	70	Cares for self; unable to carry on normal activity or to do active work.
	60	Requires occasional assistance, but is able to care for most of his personal needs.
	50	Requires considerable assistance and frequent medical care.
Unable to care for self; requires equivalent of institutional or hospital care; disease may be progressing rapidly.	40	Disabled; requires special care and assistance.
	30	Severely disabled; hospital admission is indicated although death not imminent.
	20	Very sick; hospital admission necessary; active supportive treatment necessary.
	10	Moribund; fatal processes progressing rapidly.
	0	Dead

APPENDIX 2: Mini-Mental Status Exam

Do you have any trouble with your memory?
May I ask you some questions about your memory?

		RESPONSE	SCORE	
**ORIENTATION TO TIME**				
What is the …	year?	____________________	0	1
	season?	____________________	0	1
	month of the year?	____________________	0	1
	day of the week?	____________________	0	1
	date?	____________________	0	1
**ORIENTATION TO PLACE**				
Where are we now?	state (province)?	____________________	0	1
What is the …	county (or city/town)?	____________________	0	1
	City/town (or part of city/neighborhood)?	____________________	0	1
	Building (name or type)?	____________________	0	1
	Floor of the building (room number or address)?	____________________	0	1

REGISTRATION

Listen carefully. I am going to say three words. You say them back after I stop. Ready? Here they are … APPLE [pause], PENNY [pause], TABLE [pause]. Now repeat those words back to me.

	APPLE	____________________	0	1
	PENNY	____________________	0	1
	TABLE	____________________	0	1

Now keep those words in mind. I am going to ask you to say them again in a few minutes.

ATTENTION AND CALCULATION [Serial 7s]

Now I'd like you to subtract 7 from 100. Then keep subtracting 7 from each answer until I tell you to stop.

What is 100 take away 7?	____________________	0	1
If needed, say: Keep going	____________________	0	1
If needed, say: Keep going	____________________	0	1
If needed, say: Keep going	____________________	0	1
If needed, say: Keep going	____________________	0	1

	RESPONSE	SCORE
Spell WORLD forward, then backward	D=1 L=1 R=1 O=1 W=1	__________

RECALL

What were those three words I asked you to remember?

	APPLE	________________	0	1
	PENNY	________________	0	1
	TABLE	________________	0	1

NAMING

What is this?	[Point to a pencil or pen.]	________________	0	1
What is this?	[Point to a watch.]	________________	0	1

REPETITION

Now I am going to ask you to repeat what I say. Ready? "NO IFS, ANDS, OR BUTS." Now you say that.

NO IFS, ANDS, OR BUTS.	________________	0	1

COMPREHENSION

Listen carefully because I am going to ask you to do something.
Take this paper in your right hand [pause], fold it in half [pause], and put it on the floor (or table).

TAKE IN RIGHT HAND	________________	0	1
FOLD IN HALF	________________	0	1
PUT ON FLOOR	________________	0	1

READING

Please read this and do what it says. [Show examinee the words on the stimulus form.]

CLOSE YOUR EYES	________________	0	1

WRITING

Please write a sentence.	________________	0	1

DRAWING

Please copy this design. 0 1

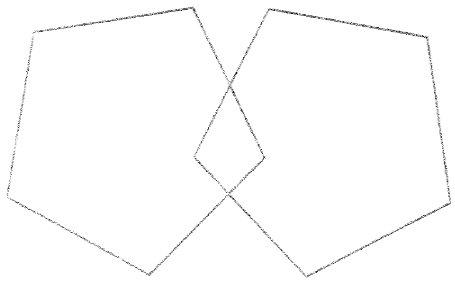

Total Score = ______

Assessment of level of consciousness

Alert Drowsy Stuporous Comatose / Unresponsive
